# EffResViT‐SE FusionNet: A Hybrid Deep Learning Framework for Accurate Classification of Coffee Leaf Diseases

**DOI:** 10.1002/fsn3.71311

**Published:** 2025-12-09

**Authors:** Bhoomika Mehta, Salil Bharany, Dalia H. Elkamchouchi, Ateeq Ur Rehman, Rahul Singh, Seada Hussen Adem

**Affiliations:** ^1^ Chitkara University Institute of Engineering and Technology Chitkara University Rajpura Punjab India; ^2^ Department of Information Technology, College of Computer and Information Sciences Princess Nourah bint Abdulrahman University Riyadh Saudi Arabia; ^3^ School of Computing Gachon University Seongnam‐si Republic of Korea; ^4^ Department of Electrical Power Adama Science and Technology University Adama Ethiopia

**Keywords:** coffee leaf disease, deep learning, EfficientNetB3, image classification, ResNet50, squeeze‐and‐excitation block, vision transformer

## Abstract

Coffee is a vital agricultural commodity that sustains millions of farmers worldwide, yet its cultivation is increasingly threatened by devastating leaf diseases such as Leaf Rust, Phoma, Cercospora, and Leaf Miner. These diseases reduce photosynthetic efficiency, cause defoliation, and ultimately lower crop yield and quality. Traditional diagnostic methods, including visual inspection and laboratory‐based tests such as PCR and ELISA, are often time‐consuming, costly, and require expert intervention, making them impractical for large‐scale use. To address these challenges, we propose EffResViT‐SE FusionNet, a novel hybrid deep learning framework that integrates EfficientNetB3 and ResNet50 enhanced with Squeeze‐and‐Excitation (SE) blocks for adaptive local feature recalibration, along with a Vision Transformer (ViT) for modeling global contextual dependencies. This fusion design effectively combines CNN‐based local feature extraction with transformer‐based long‐range attention in a unified architecture. The model was trained on a large‐scale dataset comprising 58,555 coffee leaf images distributed across five classes: Healthy (18,984), Miner (16,983), Leaf Rust (8336), Cercospora (7681), and Phoma (6571). The dataset was split into 70%, 15%, and 15% testing. Key hyperparameters included the Adam optimizer, a learning rate of 0.001, a batch size of 32, and 80 training epochs, ensuring stable convergence. Experimental results demonstrate the superior capability of the proposed model, achieving an overall classification accuracy of 99%, with precision, recall, and F1‐scores all ranging between 98% and 99% across all classes. Comparative analysis confirmed notable improvements over baseline models: ResNet50 (94% accuracy), EfficientNetB3 (95% accuracy), and standalone ViT (97% accuracy). Furthermore, ablation studies validated the critical role of SE blocks and feature fusion with the transformer in achieving optimal performance. These outcomes highlight EffResViT‐SE FusionNet as a powerful, precise, and scalable solution for early detection and classification of coffee leaf diseases, supporting timely interventions and promoting sustainable agriculture.

## Graphical abstract

This study presents EffResViT‐SE FusionNet, a deep learning‐based model that combines EfficientNetB3, ResNet50, Squeeze‐and‐Excitation (SE) blocks, and a Vision Transformer for accurate classification of coffee leaf diseases. Trained on 58,555 annotated images, the model achieved 99% accuracy and outperformed baseline architectures, offering a robust and scalable solution for early disease detection in coffee cultivation.

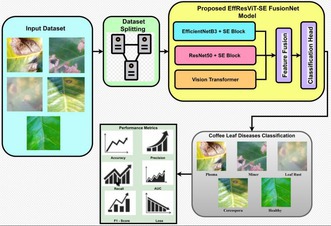

## 1 Introduction

Coffee is arguably the most valuable crop economically in the world, with several million farmers relying on its harvests to sustain themselves. Coffee cultivation, however, faces several issues, including its susceptibility to a wide range of diseases that affect both yield and quality (Tassis and Krohling 2022). Of these, the coffee leaf diseases are very harmful, causing a diminution in photo synthesis, leaf fall, and finally lower yield (Sharma et al. 2022). They are instigated by many pathogenic fungi and environmental factors, and as such, identification is important as early and accurately as possible to act on them suitably for managing and mitigating. Coffee leaf diseases are mainly caused by fungal pathogens, bacterial infections, and environmental stresses. Coffee leaf rust (Hemileia vastatrix) is one of the most prevalent fungal diseases, and it causes yellow‐orange leaf spots that result in premature defoliation. Another severe disease is phoma leaf spot (Phoma spp.), which is expressed as brown necrotic spots with a tendency to spread fast in humid weather. Cercospora leaf spot (Cercospora coffeicola) occurs as tiny brown spots with a yellow halo, infecting coffee plants when the weather is warm and moist. Coffee miner (Leucoptera coffeella) is also an insect pest that results in leaf mining, leading to a decrease in the photosynthetic capacity and vigor of the plant (Binney and Ren 2022). High humidity, temperature, and unsound agronomic practices enhance the progress of these diseases (Milke et al. 2023). The cultural, chemical, and biological control practices are traditional methods of controlling coffee leaf diseases. Proper spacing, pruning, and shade regulation through cultural practices enhance air circulation and minimize fungal spore dissemination. Chemical fungicides such as copper‐based sprays are commonly employed to manage fungal infections, although their overuse has implications for environmental and human health (Sucia et al. 2023). Biological control measures, such as the application of microbial antagonists and resistant coffee varieties, have been promoted in recent years as sustainable options. Moreover, advanced methods like remote sensing and machine learning‐based early detection systems are enhancing disease management practices (Mansouri et al. 2024). Conventional diagnosis of coffee leaf diseases is based on visual examination by farmers and agronomists. Although experienced farmers can recognize typical symptoms, the process is very subjective, time‐consuming, and susceptible to errors, especially in the initial stages of disease development (Aufar et al. 2023). Laboratory tests such as enzyme‐linked immunosorbent assay (ELISA) and polymerase chain reaction (PCR) are precise and costly and require technical expertise (Putra et al. 2022). Classification models based on deep learning provide a viable alternative where real‐time, accurate, and automated disease diagnosis with minimal human interaction is possible (Waldamichael et al. 2022). Deep learning has revolutionized plant disease classification, yielding high accuracy and resistance to detecting complex patterns in leaf images. EfficientNetB3, ResNet50, and SE Block are employed in this research to classify five coffee leaf diseases: healthy, rust, phoma, miner, and cercospora. EfficientNetB3 is a complex CNN that is highly renowned for scalability and efficiency. Constructed with compound scaling methodology, EfficientNetB3 achieves depth, width, and resolution balance and provides enhanced performance using fewer parameters than the standard CNNs. EfficientNetB3 has widely been utilized in agricultural applications due to its superior feature extraction power and computational efficiency (Hassan 2024). ResNet50 (Residual Network of 50 layers) is a deep CNN design that solves the vanishing gradients issue through residual connections. Using identity mappings, ResNet50 enables deep networks to learn strong features without any loss in performance. This model has achieved exceptional success in plant disease classification because of its ability to extract deep features and invariance to lighting and leaf variations (Jepkoech 2023). SE Block is an operation of recalibrating a feature that enhances the representational power of CNN. By dynamically adjusting channel‐wise feature responses, SE Block enables the network to give more attention to important regions of the image, and thus the classification accuracy is better. Integration of SE Block with CNN architectures like ResNet50 and EfficientNetB3 enhances the efficacy of disease classification by increasing scrutiny to patterns for the disease (Ocaña‐Zuñiga et al. 2024). Despite a huge increase in coffee disease classification, a few challenges persist. Current disease identification techniques are slow, subjective, and not effective in early detection (Kulkarni et al. 2023). Current deep learning‐based models, although powerful, tend to overfit because of restricted annotated data, thus being challenging for real‐world deployment. In addition, inconsistencies in leaf appearance caused by lighting, occlusions, and superimposition of symptoms hinder precise classification (Hitimana et al. 2023). To overcome these limitations, this study suggests a strong deep learning‐based classification system using EfficientNetB3, ResNet50, and SE Block for enhanced accuracy and generalization in the detection of coffee leaf disease (Alirezazadeh et al. 2023).

While previous research has explored individual CNN architectures or standalone transformer models for plant disease classification, our work introduces a novel hybrid model that leverages the complementary strengths of convolutional networks and Vision Transformers. By integrating ResNet50 and EfficientNetB3 (with SE Blocks) for deep spatial feature extraction and ViT for long‐range contextual learning, the proposed EffResViT‐SE FusionNet offers a powerful fusion‐based framework. This design enhances attention to both fine‐grained disease patterns and global image structure, addressing the limitations of overfitting, poor generalization, and limited feature sensitivity in prior works. The motivation for this research stems from the increasing need for reliable and scalable solutions for automatic plant disease detection, particularly in coffee cultivation, where early diagnosis is critical for minimizing yield loss. Traditional deep learning methods, especially CNNs, are effective at extracting local features but often struggle with capturing global context and generalizing across visually similar disease classes. On the other hand, Vision Transformers can model long‐range dependencies but typically require large datasets and suffer from overfitting when used in isolation. Therefore, we were motivated to develop a hybrid deep learning framework that combines the strengths of CNNs (for local pattern recognition), SE blocks (for feature recalibration), and ViTs (for global attention) into a single, unified architecture. This fusion aims to achieve higher classification accuracy, improved feature sensitivity, and better generalization performance across complex, real‐world agricultural datasets. Existing algorithms for plant disease classification, especially CNN‐based architectures such as ResNet and EfficientNet, have shown good performance in capturing local spatial features like color, texture, and edges. However, they often lack the ability to capture long‐range contextual relationships, making them less effective when disease patterns span larger regions of the leaf or when background noise interferes. Conversely, Vision Transformers (ViTs) are capable of modeling global dependencies but are typically data‐hungry, computationally expensive, and prone to overfitting on limited datasets, especially in agricultural domains where fine‐grained disease features are subtle and inter‐class similarity is high. To address these shortcomings, the proposed EffResViT‐SE FusionNet integrates CNN‐based architectures (ResNet50, EfficientNetB3) with SE attention blocks and a Vision Transformer. This hybrid model combines the strengths of CNNs for extracting detailed local features, SE blocks for adaptive channel attention, and ViTs for capturing high‐level semantic context. By fusing these feature streams, our model achieves greater robustness, higher accuracy, and better generalization than any single architecture alone, effectively overcoming the individual limitations of existing methods.

The major contributions of the work are as follows:

- ResNet50 with SE Block was used to leverage deep residual learning for effective hierarchical feature extraction while the SE Block enhanced the model's ability to focus on disease‐specific channels, improving classification of fine‐grained leaf symptoms.
- EfficientNetB3 with SE Block was chosen because of its compound scaling property, where high accuracy was achieved using fewer parameters, and the SE Block also improved its feature maps by emphasizing informative patterns and removing noise, hence being effective for resource‐efficient learning.
- ViT was utilized to represent global dependencies and contextual relations in leaf images through self‐attention mechanisms, which bypassed the shortcoming of CNNs in modeling long‐range interactions within the image.
- Ensemble Model (EffResViT‐SE FusionNet) was created to combine spatial and contextual features obtained by ResNet50‐SE, EfficientNetB3‐SE, and ViT to create a strong hybrid architecture that attained a classification accuracy of 99% and outperformed separate models in all the metrics.

## 2 Literature Review

Recent advances in deep learning have driven remarkable progress in agricultural image analysis, enabling automated, accurate recognition of plant diseases. Researchers have adopted a spectrum of architectures from conventional convolutional networks to hybrid and transformer‐based systems each contributing unique strengths in precision, recall, computational efficiency, and robustness as shown in Table 1.

**TABLE 1:** Literature review.

References, year of publishing	Name of journal/conference/workshop/book chapter/LNCS	Technique	Number of images	Results
Karia et al. (2025)	African Journal of Empirical Research, 2025	DenseNet201	9031 images	Accuracy: 97.49%
Gedela (2025)	i‐Manager's Journal on Artificial Intelligence & Machine Learning (JAIM)	DenseNet & ResNet	—	Accuracy: 82.3%
Nawaz et al. (2024)	Expert Systems with Applications, 2024	CoffeeNet (ResNet‐50 model with spatial‐channel attention)	—	Accuracy: 98.54%
Hasan et al. (2023)	Plants, 2023	ResNet50 classifier	3003 images	Accuracy: 98%
Chavarro et al. (2023)	Applied Sciences, 2023	DenseNet201	5337 images	Accuracy: 94.80%
Prates et al. (2024)	International Symposium on Neural Networks, 2024	MobileNet CNN model	—	F1 score of 95.414%
Fragoso et al. (2025)	Applied Sciences, 2025	YOLOv8, YOLOv9, YOLOv10, and YOLOv11	1747 images	YOLOv8s: mAP: 54.5% F1‐Score: 54% Recall: 93%
Araaf et al. (2024)	Sensors, 2024	YOLOv5 YOLOv8	2024 images	Accuracy: (YOLOv5): 69% (YOLOv8): 70.2%
Salamai and Al‐Nami (2023)	Sustainability (MDPI)	NAML	1747 full coffee‐leaf images	Accuracy: 96.15%–96.69% Precision: 97.2% For severity: 90%
Novtahaning et al. (2022)	Agriculture, 2022	EfficientNet‐B0, ResNet‐152, VGG‐16, InceptionV3, Xception, MobileNetV2, DenseNet201, InceptionResNetV2, and NasNetMobile	1300 images	Accuracy: 97.31%
Yamashita and Leite (2023)	Smart Agricultural Technology, 2023	Cascade and Single‐Stage architectures	6414 images	Cascade Architecture Accuracy: 98% Single‐Stage Architecture Accuracy: 96%
Vilela et al. (2024)	AgriEngineering, 2024	SVM and SGD models	40 images per farm	F‐measure values of 91.5% and 87.5%
Soares et al. (2022)	Agronomy, 2022	Support Vector Machine (SVM) for classification	320 coffee seedlings images	Accuracy: 80%
Marin et al. (2021)	Computers and Electronics in Agriculture, 2021	LMT	—	F‐measure values of 91.5% and 87.5%
Orlando et al. (2024)	Agriculture, 2024	Threshold‐Based Detection Approach	160 coffee leaves images	—
Yebasse et al. (2021)	Plants (MDPI), 2021	DL (Guided & Naïve)	1560 images	Guided Accuracy: 98% Naïve accuracy: 77%
Esgario et al. (2020)	Computers and Electronics in Agriculture, 2020	ResNet50	1747 images	Biotic Stress Classification Accuracy: 95.24% Severity Estimation Accuracy: 86.51% Symptom‐Only Classification: > 97% Accuracy
Sucia et al. (2023)	Kinetik: Game Technology, Information System, Computer Network, Computing, Electronics, and Control	VGG19 & ResNet50	1560 images	VGG19 F1‐score: 90% Accuracy: 90% ResNet50: Accuracy: 81% Precision: 85% Recall: 77% F1‐score: 81%
Sorte et al. (2019)	Procedia Computer Science, 2019	CNN (AlexNet)	1560 images	Kappa coefficient: 97% Sensitivity: 98%

Deep convolutional models remain the most prevalent approach for coffee‐leaf disease recognition. Karia et al. (2025) employed DenseNet201 achieving 97.49% accuracy on 9031 images, validating DenseNet's strong feature reuse capability. Gedela (2025) combined DenseNet and ResNet obtaining 82.3% accuracy, illustrating that model depth alone does not guarantee stability on imbalanced data. Nawaz et al. (2024) enhanced ResNet‐50 with spatial channel attention (CoffeeNet) and attained 98.54% accuracy. Hasan et al. (2023) similarly fine‐tuned ResNet50 on 3003 images to reach 98% accuracy, confirming transfer learning efficiency, while Chavarro et al. (2023) reported 94.80% accuracy using DenseNet201 but without recall or F1 disclosure, limiting assessment of minority class reliability. These CNN studies highlight high discriminative power yet limited global context awareness.

To address speed and deployment, compact networks such as MobileNet and YOLO have been examined. Prates et al. (2024) used MobileNet CNN, producing an F1 of 95.414% with minimal parameters. Fragoso et al. (2025) proposed a YOLO‐based detector for coffee‐leaf diseases and pests, achieving mAP 54.5%, F1 54%, and recall 93%, revealing strong detection sensitivity but moderate precision. Araaf et al. (2024) compared YOLOv5 and YOLOv8, obtaining 69% and 70.2% accuracy, respectively adequate for rapid recognition yet inferior to heavier CNNs in fine‐grained discrimination. These studies demonstrate that one‐stage detectors trade accuracy for real‐time capability.

Salamai and Al‐Nami (2023) introduced a meta learning NAML framework, attaining 96.15%–96.69% accuracy and 97.2% precision, reflecting improved generalization with limited data. Novtahaning et al. (2022) evaluated multiple CNN backbones: EfficientNet‐B0, ResNet‐152, VGG‐16, InceptionV3, Xception, MobileNetV2, DenseNet201, and InceptionResNetV2 and reported peak accuracy of 97.31% using EfficientNet‐B0 with favorable parameter efficiency. Yamashita and Leite (2023) compared cascade and single‐stage architectures with accuracies of 98% and 96%, noting that multi‐stage pipelines enhance recall and robustness. Collectively, these works reveal a shift toward hybrid and attention‐integrated frameworks that balance precision, recall, and computational load.

Earlier studies utilized shallow models for benchmark comparison. Vilela et al. (2024) employed SVM and SGD classifiers, achieving F‐measures of 91.5% and 87.5%. Soares et al. (2022) used SVM for 320 coffee‐seedling classification, recording 80% accuracy. Marin et al. (2021) applied LMT, reaching F‐measures of 91.5% and 87.5%. Although these methods are computationally light, they lack scalability and spatial feature extraction capability compared with CNNs.

Orlando et al. (2024) proposed a threshold‐based detector on 160 images for preliminary classification, while Yebasse et al. (2021) compared guided and naive deep‐learning strategies on 1560 images, obtaining guided accuracy of 98% and 77% naive, confirming the value of supervised guidance. Esgario et al. (2020) fine‐tuned ResNet50 for biotic‐stress classification, achieving 95.24% accuracy, with severity estimation of 86.51% and symptom‐only classification > 97%, demonstrating that deep networks can capture stress severity beyond categorical labels.

Sucia et al. (2023) benchmarked VGG19 and ResNet50 on 1560 images. VGG19 reached 90% accuracy and F1, outperforming ResNet50 with 81% accuracy, 85% precision, 77% recall. Sorte et al. (2019) employed AlexNet on 1560 images and achieved Kappa 97% and sensitivity 98%, proving reliability in binary plant‐disease recognition. These comparisons illustrate that classical CNNs still perform competitively when optimized on small, homogeneous datasets.

Across the reviewed studies, accuracy ranges from 70% to 98%, yet precision, recall, and F1 are inconsistently reported, limiting holistic assessment of diagnostic reliability. CNN‐based models provide superior local feature learning but struggle to model long‐range dependencies; one‐stage detectors enhance speed at the expense of precision, while meta‐learning and transformer‐augmented methods offer balanced metrics but often demand extensive computation. Moreover, prior datasets seldom capture variability in leaf age, illumination, and background conditions, restricting field generalization. Addressing these gaps, the proposed EffResViT‐SE FusionNet integrates ResNet50 and EfficientNet B3 backbones with Squeeze‐and‐Excitation channel attention and a Vision Transformer module to fuse local texture and global context. The resulting hybrid model achieves balanced performance across accuracy 99%, precision of 0.99, recall of 0.99, and F1 of 0.99, establishing a robust foundation for scalable, interpretable coffee leaf disease classification.

## 3 Methods and Material

The study utilizes a coffee leaf diseases dataset of 58,555 images, divided into Rust, Phoma, Miner, Cercospora and Healthy for training, validation, and testing. The proposed EffResViT‐SE FusionNet Model integrates ResNet50, EfficientNetB3 with SE Block and ViT to refine feature extraction and multi‐scale convolutional blocks to classify coffee leaf diseases. To achieve a stable and efficient learning process, Adam optimizer, learning rate (getting less than 0.001), batch size (32), and epochs (80) are trained in the model.

### 3.1 Input Dataset

**FIGURE 1:**
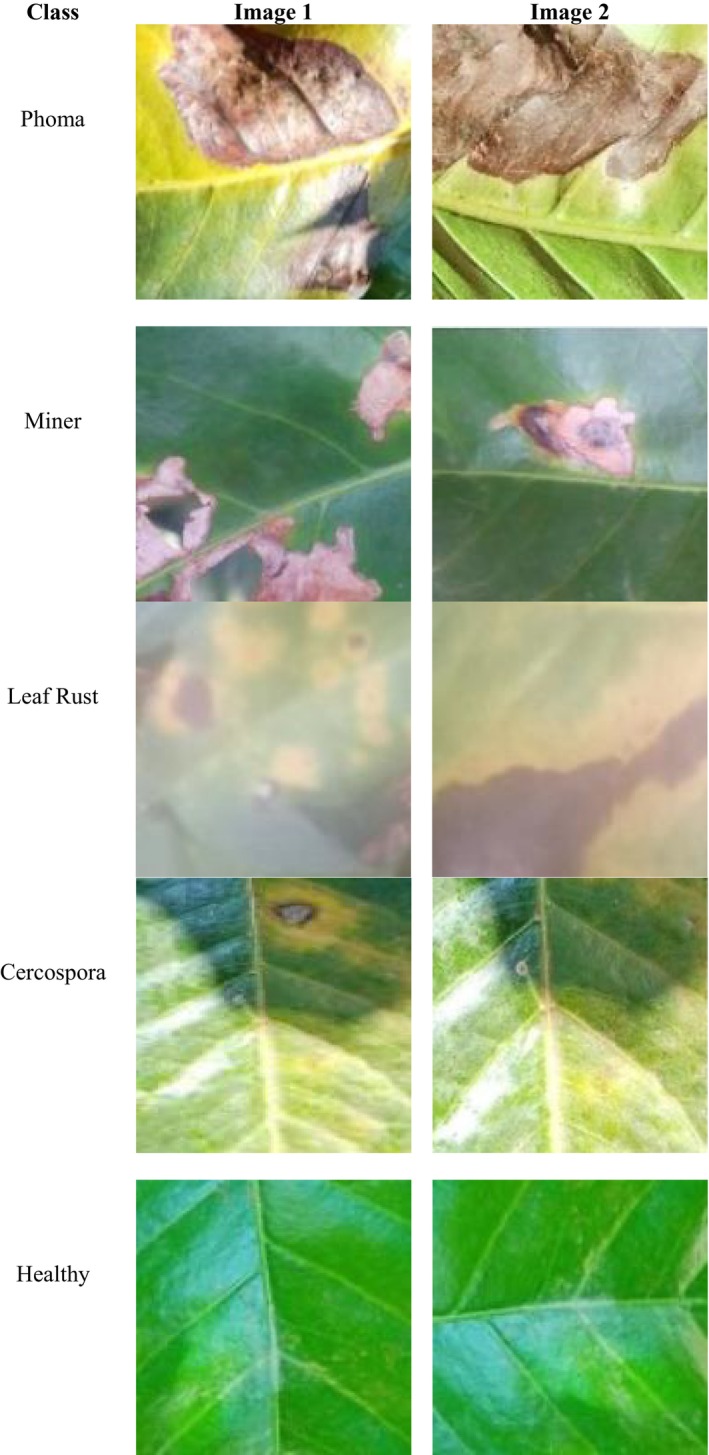
Input dataset.

This study utilized the publicly available dataset from the Kaggle repository. The dataset consists of 58,555 images across five categories, each representing a distinct class of coffee leaf conditions. The dataset is structured into directories, including Healthy (18,984 files), which contains images of unaffected coffee leaves, serving as the baseline for comparison. The Cercospora category (7681 files) includes images of leaves affected by Cercospora leaf spot, a fungal disease that causes brown lesions. The Leaf Rust directory (8336 files) comprises images depicting coffee leaves infected with Hemileia vastatrix, a common fungal disease causing yellow‐orange powdery spots. The Miner category (16,983 files) contains images of leaves damaged by coffee leaf miners, which create characteristic serpentine tunnels due to larval feeding. Finally, the Phoma class (6571 images) contains images depicting symptoms of Phoma leaf spot, a fungal disease that causes dark necrotic lesions as depicted in Figure 1. These images are resized to 224 × 224 × 3. These diseases were chosen because they are the most common and economically significant threats to coffee production, collectively responsible for severe yield reduction and quality loss across coffee‐growing regions worldwide. Leaf Rust, caused by Hemileia vastatrix, is historically the most devastating fungal disease, while Phoma and Cercospora are also major fungal infections that severely affect leaf tissue and crop health. Miner, in contrast, represents an insect‐related stressor, introducing additional variability in symptom patterns and making the classification task more challenging. This diverse selection ensures that the dataset covers both fungal and insect‐related diseases, allowing the model to learn a wide range of feature patterns. Other diseases, such as coffee leaf spot, were not included due to their lower prevalence in the available dataset and the limited number of annotated images, which could have caused class imbalance and reduced the robustness of the model. This dataset offers a rich and well‐balanced set of images, which is necessary for training deep learning models to classify coffee leaf diseases accurately. Besides the categorical attempt of organizing the dataset, the images include coffee leaves of different age and physiological status of young, tender leaves as well as completely mature and senescent leaves. The variety of types of visual progression of the disease guarantees the diversity of the dataset because at the early stages of infection, the disease looks like some chlorotic spots which are barely noticeable on the younger leaves, whereas mature leaves show characteristic necrotic spots or miner tracks. The photographs were gathered in the heterogeneous field conditions such as daytime and shady conditions to capture the scenario of realistic variability in illumination, moisture, and background textures. The data is a combination of samples from various coffee plantations and Internet reserves that provide their variety, guaranteeing regionality and morphological abundance.

### 3.2 Dataset Splitting

The dataset is split into training 70%, testing 15%, and validation (15%) sets with a balanced distribution for training and evaluation of the model as represented in Table 2. The Healthy class is the most populous with 13,289 images for training, 2848 for testing, and 2847 for validation; then the Miner class with 11,888, 2547, and 2548 images, respectively. The Cercospora class has 5377 training images, 1152 test images, and 1152 validation images, whereas the Leaf Rust class has 5835, 1250, and 1251 images for each of the sets. The Phoma class has the lowest number with 4600 images for training, 986 for testing, and 985 for validation. This structured dataset split ensures the deep learning model is well trained without overfitting.

**TABLE 2:** Dataset splitting.

Class	Training (70%)	Testing (15%)	Validation (15%)
Cercospora	5377	1152	1152
Healthy	13,289	2848	2847
Leaf Rust	5835	1250	1251
Miner	11,888	2547	2548
Phoma	4600	986	985

### 3.3 Proposed EffResViT‐SE FusionNet Model

**FIGURE 2:**
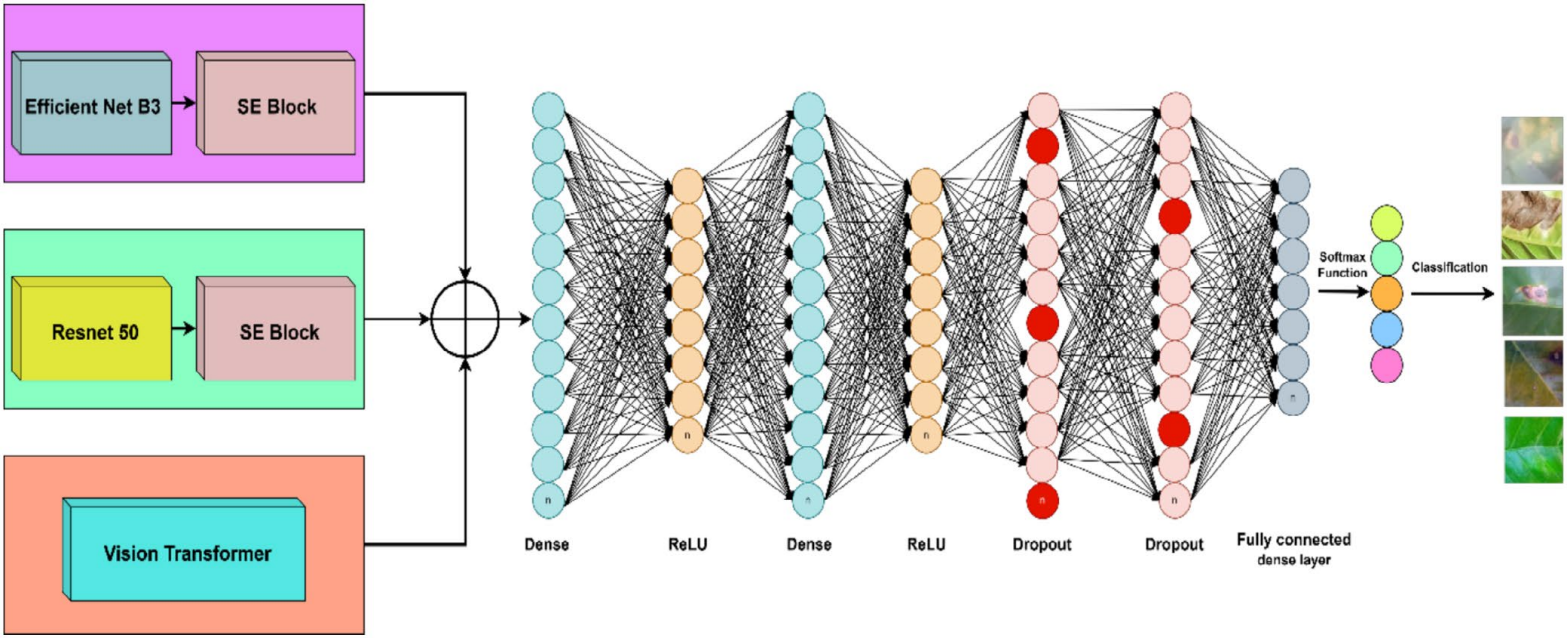
Proposed EffResViT‐SE FusionNet Model.

The Figure 2 illustrates a multi‐stream EfficientNetB3‐based deep learning model, ResNet50, and a ViT for feature extraction with a concatenation‐based classification architecture. One of the three models is employed for every input image, with EfficientNet B3 and ResNet50 augmented by an SE block for enhanced feature recalibration. The ViT learns features by mapping image patches into token embeddings prior to their passage through a transformer encoder. The feature maps learned from each of the three architectures are stacked and inputted into a multilayered fully connected neural network with several dense layers to learn features and classify. The last classification layer provides predictions on various leaf disease classes, represented by the right‐hand side images. This merging approach strengthens the model to detect both spatial and contextual characteristics, resulting in accurate and strong leaf disease classification.

The novelty of the proposed framework lies not only in the use of well‐established models such as ResNet50, EfficientNetB3, and the ViT, but more importantly in the way these architectures are integrated into a unified design. Specifically, ResNet50 is enhanced with an SE block after global average pooling (GAP), which recalibrates feature channels dynamically to emphasize disease‐specific patterns such as necrotic spots or miner trails while suppressing background noise. Similarly, EfficientNetB3, known for its compound scaling that balances depth, width, and resolution, is augmented with SE blocks between GAP layers, thereby improving representational capacity without significantly increasing the number of parameters. This modification allows EfficientNetB3‐SE to efficiently capture fine‐grained visual cues relevant to coffee leaf diseases. In parallel, the ViT processes input images as patch embeddings and employs multi‐head self‐attention to model long‐range dependencies, effectively capturing global contextual information that CNNs typically fail to address. The outputs from ResNet50‐SE, EfficientNetB3‐SE, and ViT are then concatenated into a unified feature vector, which is subsequently passed through fully connected layers with dropout regularization. This fusion strategy combines local hierarchical features from ResNet50‐SE, parameter‐efficient representations from EfficientNetB3‐SE, and global dependencies from ViT, resulting in richer and more discriminative feature learning than any single architecture can achieve. This structured integration of SE‐enhanced CNNs with a transformer branch forms the core contribution of the study, with subsequent subsections (3.3.1–3.3.5) providing detailed explanations of each component and the overall fusion pipeline.

#### 3.3.1 Squeeze and Excitation Block

**FIGURE 3:**
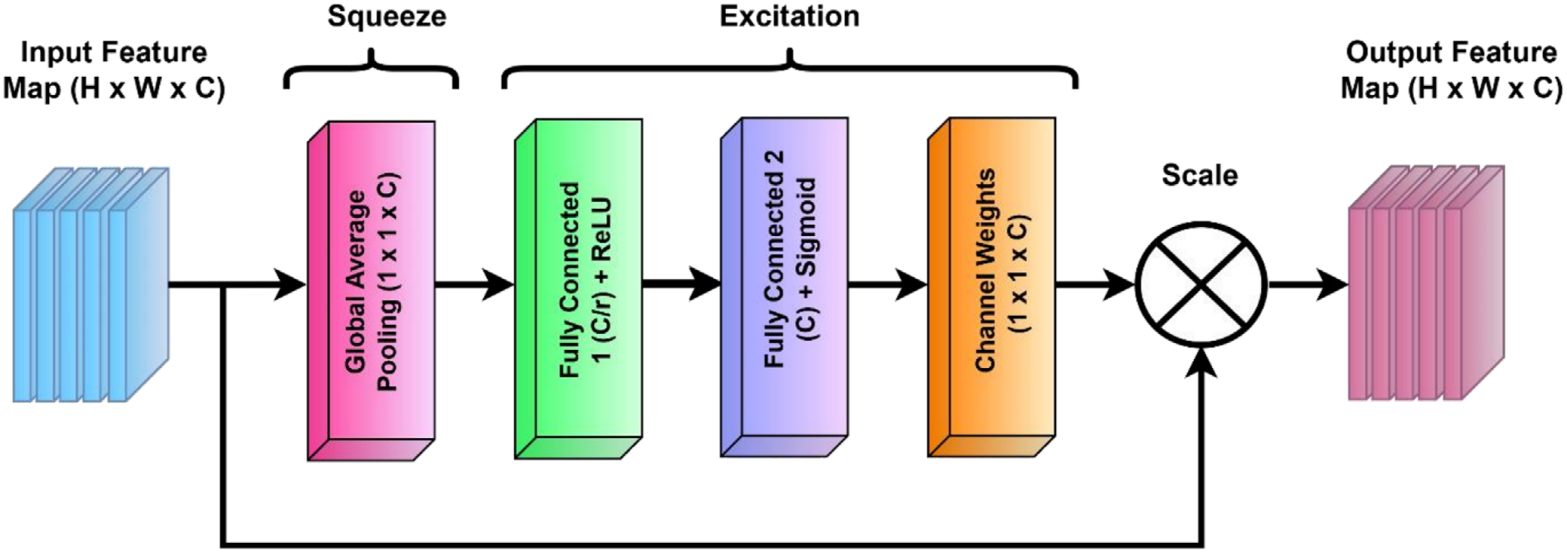
Squeeze and excitation block.

The Figure 3 illustrates the architecture of a Squeeze‐and‐Excitation (SE) block, a channel attention mechanism designed to enhance feature representations in CNNs. In the squeeze stage, GAP reduces each feature map into a compact 1 × 1 × C descriptor, summarizing global spatial information across channels. In the excitation stage, this descriptor is first passed through a Fully Connected (FC) layer with dimensionality reduction by a ratio and a ReLU activation, followed by a second FC layer that restores the original channel size and applies a Sigmoid activation to produce channel attention weights between 0 and 1. These learned weights (1 × 1 × C) are then broadcast and multiplied element‐wise with the original feature maps, thereby selectively emphasizing informative channels and suppressing less useful ones. Through this squeeze‐and‐excitation mechanism, the network adaptively recalibrates channel‐wise features, improving its ability to highlight disease‐relevant patterns while minimizing background noise.

The SE Block, illustrated in Figure 3, is a channel attention mechanism that adaptively recalibrates feature responses to enhance the representational power of CNNs. The SE Block operates through two main stages: Squeeze and Excitation. In the Squeeze stage, Global Average Pooling (GAP) aggregates spatial information across each channel to generate a channel descriptor $S_{c}$ as shown in (Equation 1):

(1) $$S_{c}=\frac{1}{H\times W}\;\sum_{i=1}^{H}\sum_{j=1}^{W}X_{c}\left(i,j\right)$$

where $X_{c}\left(i,j\right)$ denotes the activation at spatial location $\left(i,j\right)$ of channel C and H and W are the height and width of the feature map.

In the Excitation stage, the squeezed vector $S_{c}$ passes through two fully connected (FC) layers. The first FC layer reduces the dimensionality by a ratio r and applies the ReLU activation, while the second restores the channel dimension and applies the Sigmoid activation to generate the excitation weights E, as shown in (Equation 2):

(2) $$E=\sigma^{\prime }\left(W_{2}\sigma (W_{1}S_{c}\right))$$

where $W_{1}\in R^{C\times \frac{C}{r}}$ and $W_{2}\in R^{C\times \frac{C}{r}}$ are the learnable weight matrices, $\sigma \left(\cdot \right)=\max\left(0,\cdot \right)$ denotes the ReLU activation, and $\sigma^{\prime }\left(\cdot \right)=\frac{1}{1+e^{-x}}$ represents the Sigmoid activation.

Finally, the recalibrated output feature map $X^{\prime }$ is obtained by channel‐wise multiplication of the original feature map X and the excitation weights E, as shown in (Equation 3):

(3) $$X^{\prime }=X.E$$

#### 3.3.2 Integrated SE Block With Fine‐Tuned ResNet50 Model

**FIGURE 4:**
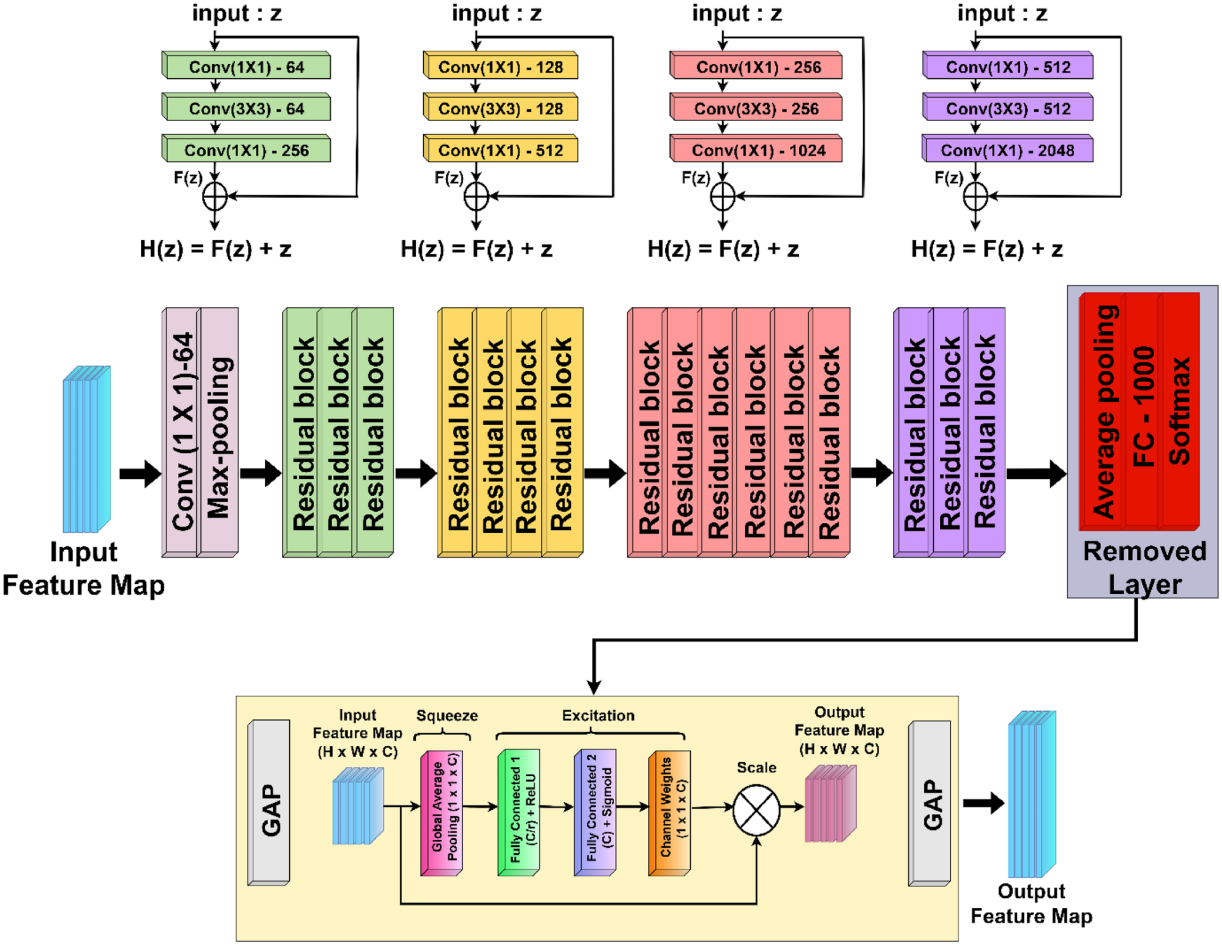
Modified ResNet50 architecture with integrated SE blocks.

The Figure 4 illustrates the architecture of ResNet‐50 with modifications incorporating SE Blocks. It begins with a 7 × 7 convolution and max pooling, followed by multiple residual blocks that form the backbone of ResNet‐50. Each residual block consists of 1 × 1, 3 × 3, and 1 × 1 convolutional layers, progressively increasing the number of filters (64, 128, 256, 512, and 2048). The standard ResNet‐50 architecture originally concludes with GAP, a FC layer with 1000 neurons, and a SoftMax activation. However, in this modified version, the final FC layer and SoftMax activation are removed, and an SE block is added after GAP. This integration of SE blocks enhances channel‐wise feature recalibration, allowing the model to prioritize important features dynamically. This modified ResNet‐50 improves performance by refining feature representations, making it effective in tasks like classification and object recognition.

The given image represents a modified ResNet50 architecture with a SE Block. It shows the replacement of the original FC layer with an SE block to refine feature extraction.

The input image X passes through an initial convolution layer followed by max‐pooling as shown in (Equation 4) and (Equation 5):

(4) $$X_{1}=\text{Conv}\left(X,W_{1}\right)+B_{1}$$

(5) $$X_{2}=\text{MaxPool}\left(X_{1}\right)$$

where X is the input image, $W_{1}\;\text{and}\;B_{1}$ are the weights and biases of the convolution layer and $\text{MaxPool}\left(.\right)$ is the max‐pooling operation.

Residual learning is maintained through shortcut connections, where the output of each stage is shown in (Equation 6):

(6) $$X_{l+1}=X_{l}+F\left(X_{l},W_{l}\right)$$

After the final residual stage $\left(X_{6}\right)$, the SE Block is integrated to perform channel recalibration.

The squeeze operation applies GAP across spatial dimensions as shown in (Equation 7):

(7) $$S_{c}=\frac{1}{H\times W}\sum_{i=1}^{H}\sum_{j=1}^{W}X_{6},c\left(i,j\right)$$

This is followed by excitation through two fully connected layers as shown in (Equation 8):

(8) $$E=\sigma \left(W_{2}\text{ReLU}\;\left(W_{1}S\right)\right)$$

Finally, channel‐wise scaling recalibrates the feature maps as shown in (Equation 9):

(9) $$X^{\prime }=X_{6}.E$$

A final GAP and Softmax layer produces the class probabilities as shown in (Equation 10):

(10) $$Y=\text{Softmax}\left(\mathrm{GAP}\left(X^{\prime }\right)\right)$$

The entire ResNet50 + SE Block process can be compactly represented as shown in (Equation 11):

(11) $$Y=\text{Softmax}\left(\mathrm{GAP}\left(X_{6}\cdot \sigma \left(W_{2}\text{ReLU}\left(W_{1}S\right)\right)\right)\right)$$

#### 3.3.3 Integrated SE Block With Fine‐Tuned EfficientNetB3 Model

**FIGURE 5:**
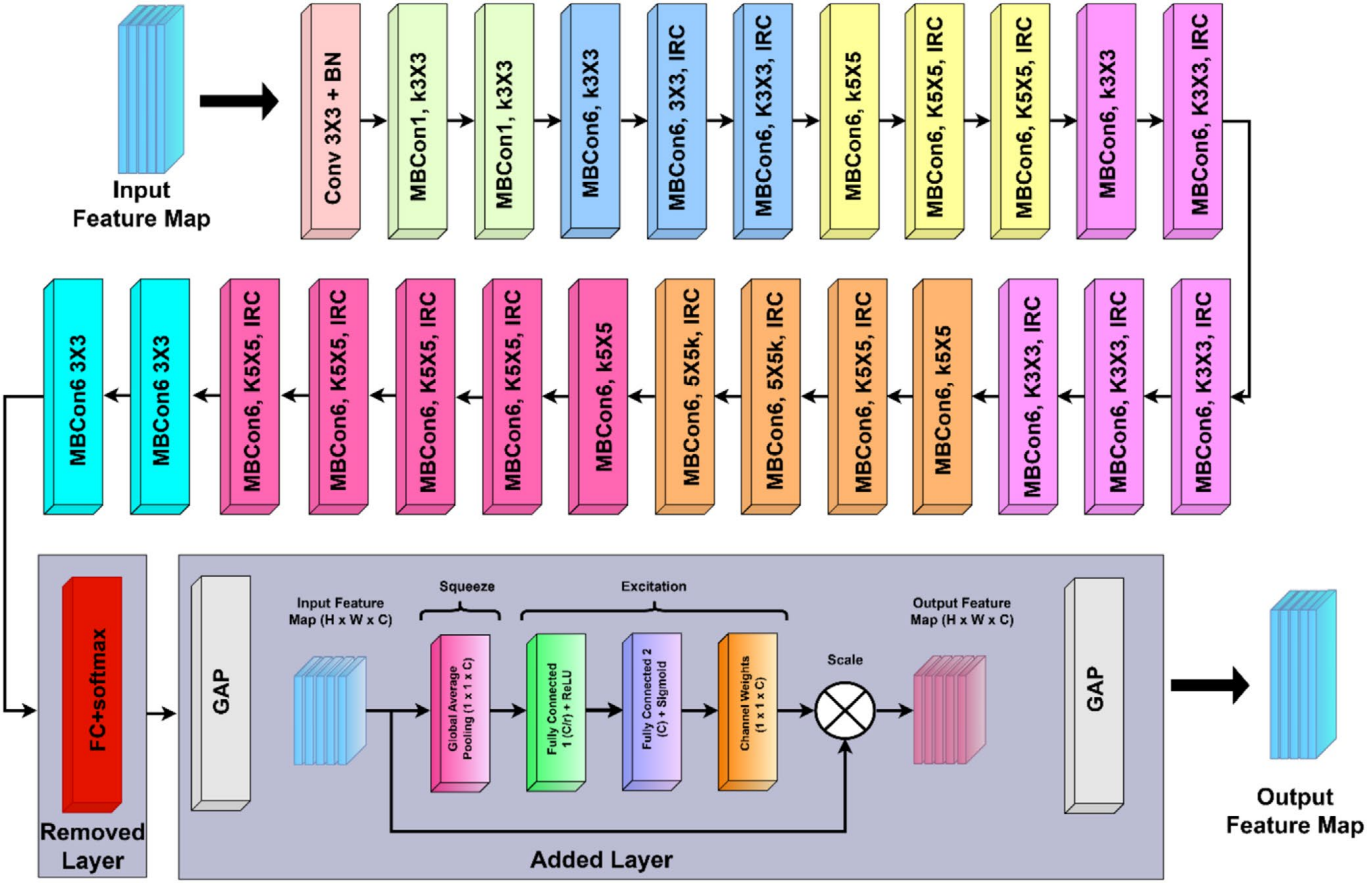
Modified EfficientNetB3 architecture with integrated SE blocks.

The Figure 5 represents the architecture of EfficientNet‐B3 with modifications integrating an SE Block. It begins with a 3 × 3 convolution, is then followed by a BN, and then sequentially by several Mobile Inverted Bottleneck Convolutions (MBConvs) that have different sizes of kernels (3 × 3, 5 × 5) and expansion ratios. These MBConvs utilize depthwise separable convolutions and inverted residual connections (IRC) to enhance efficiency and feature extraction. The standard EfficientNet‐B3 architecture originally ends with GAP, a FC layer, and a softmax activation function, but in this modified version, the FC layer and softmax activation are removed, and an SE Block is added between two GAP layers. This enhancement enables channel‐wise feature recalibration, allowing the model to focus on important features dynamically, thereby improving classification performance while maintaining computational efficiency. (3 × 3, 5 × 5).

The image represents the modified EfficientNet‐B3 architecture integrated with a SE Block. This integration enhances classification performance without increasing model size significantly.

The input image X undergoes an initial convolution, normalization, and activation as shown in (Equation 12):

(12) $$X_{1}=\text{ReLU}6\left(\mathrm{BN}\left({\text{Conv}}_{3\times 3}\left(X\right)\right)\right)$$

Subsequently, a sequence of MBConv layers with different kernel sizes and expansion factors is applied as shown in (Equation 13):

(13) $$X_{n+1}=\text{MBConv}\left(X_{n},k,\text{expand}\right)$$

where $k\in \left\{3\times 3,5\times 5\right\}$ and $\text{expand}\in \left\{1,6\right\}$ representing the variations in kernel size and expansion ratio across stages. Each MBConv employs depthwise separable convolutions, batch normalization, and inverted residual connections for efficient feature extraction.

After the final MBConv layer output $X_{12}$, the original structure is replaced with an SE Block for channel attention, followed by a final GAP and Softmax classifier, as expressed in compact form as shown in (Equation 14):

(14) $$Y=\text{Softmax}\left(\mathrm{GAP}\left(\mathrm{SE}\left(\mathrm{GAP}\left(X_{12}\right)\right)\right)\right)$$

Here, the SE Block performs channel recalibration as shown in (Equation 15):

(15) $$\mathrm{SE}\left(\mathrm{GAP}\left(X_{12}\right)\right)=X_{12}\cdot \sigma \left(W_{2}\;\text{ReLU}\left(W_{1}S\right)\right)$$

where S is the squeezed descriptor from $\mathrm{GAP},W_{1}\;\text{and}\;W_{2}$ are learnable parameters, and $\sigma \left(\cdot \right)$ is the Sigmoid activation. The output Y represents the class probability distribution generated by the final Softmax layer.

#### 3.3.4 Feature Extraction Using Vision Transformer

**FIGURE 6:**
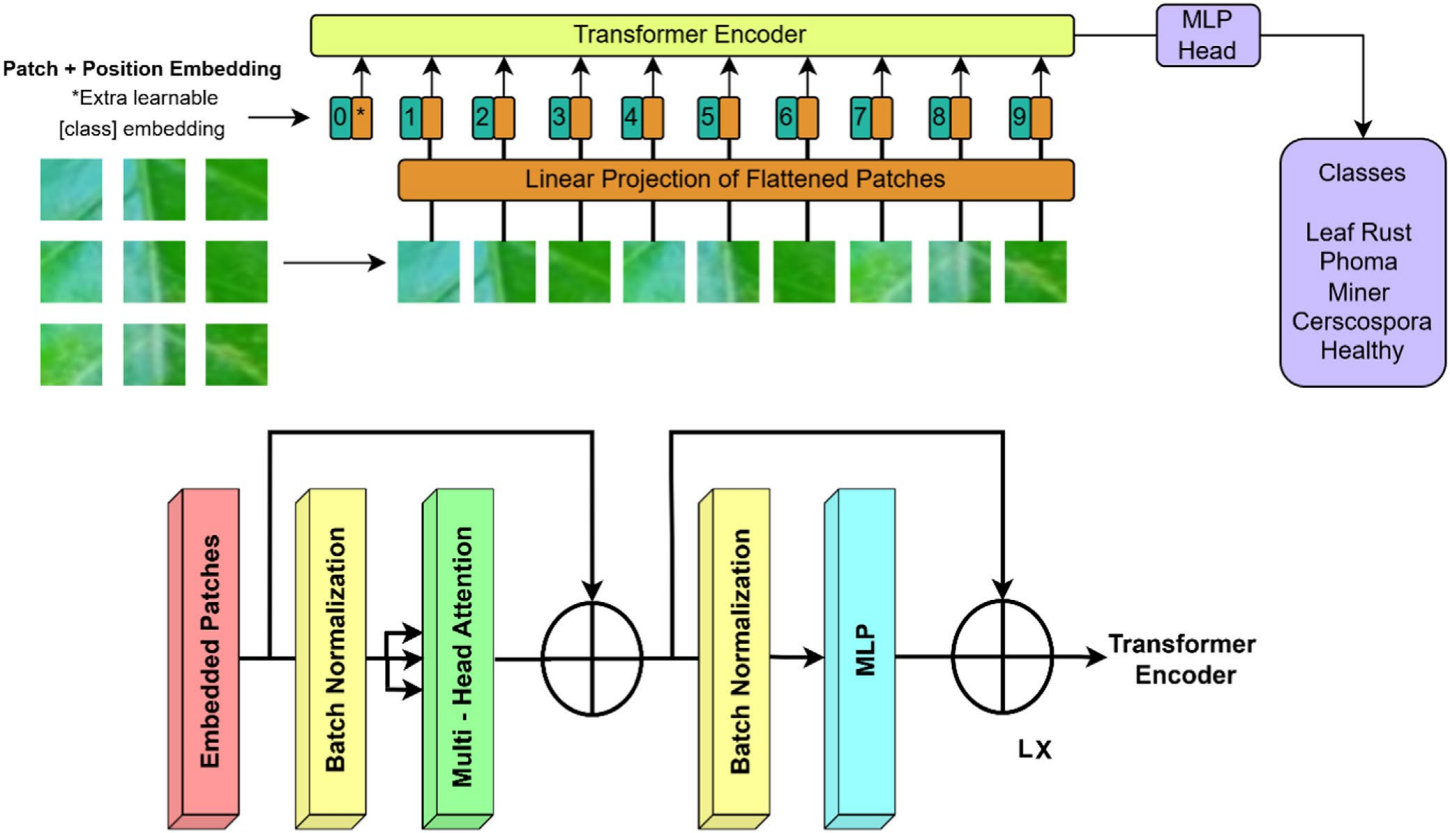
Architecture of vision transformer for feature extraction.

The Figure 6 illustrates the architecture of ViT applied to coffee leaf disease classification. ViT is a deep learning model that classifies images using a sequence learning idea commonly used by natural language processing models (transformers). In ViT, each input image (e.g., 224 × 224 × 3) is first split into a sequence of fixed‐size non‐overlapping patches (typically 16 × 16), then flattened and linearly projected into embedding vectors, as opposed to processing the whole image grid directly. There is an insertion of a special classification token ([CLS]) to the sequence, and learnable positional encodings are used to preserve structural information. It feeds this sequence into several transformer encoder blocks which include multi‐head self‐attention and feed‐forward networks through which the model learns global dependencies across the whole image. The system does classification based on the output corresponding to the [CLS] token. ViT can supplement CNN‐based building blocks in the proposed architecture, since it learns long‐range contextual correlations between regions of coffee leaves, which play an essential role in detecting the presence of complex or non‐contiguous signs of disease in a coffee leaf. The first picture is the ViT pipeline in which an input image is broken into patches of fixed size and then linearly projected into embeddings and fed into a Transformer Encoder. A class token (0*) is appended to the sequence, and the encoded representations are processed by a Multi‐Layer Perceptron (MLP) Head to classify the leaf as Leaf Rust, Phoma, Miner, Cercospora, or Healthy. The second image details the Transformer Encoder Block, which consists of patch embeddings, batch normalization, multi‐head self‐attention, and a feedforward MLP. In our implementation, each input image of size 224 × 224 × 3 is divided into non‐overlapping patches of size 16 × 16, resulting in a total of *N* = 196 patches (since 224/16 = 14 patches per dimension and 14 × 14 = 196). Each patch is flattened into a 1D vector of length *p* = 768 using a linear projection. The stride used is equal to the patch size (16), ensuring non‐overlapping token generation. Residual connections and layer normalization are applied after each module to improve gradient flow. This architecture helps ViT to capture long‐range dependencies in images effectively, and thus can be a very useful alternative to CNNs for detecting diseases in leaves.

#### 3.3.5 Feature Fusion and Classification

**FIGURE 7:**
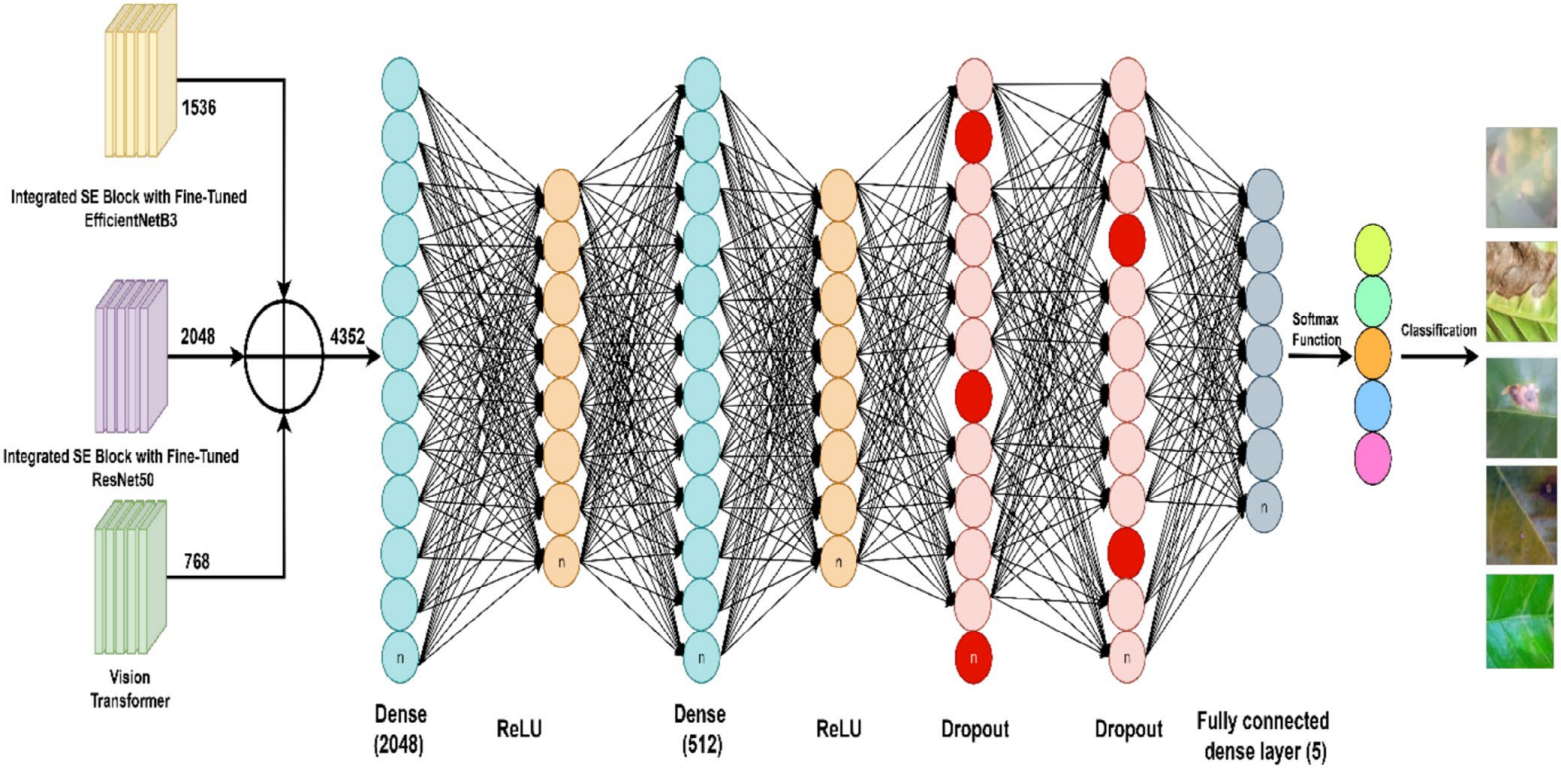
EffResViT‐SE FusionNet: integrated feature extraction and classification.

The Figure 7 represents a concatenation‐based deep learning architecture designed for multi‐source feature fusion in an image classification task. It consists of three different input streams, each processed through separate feature extraction networks before their outputs are concatenated at an intermediate layer. The concatenated feature representation is then passed through multiple FC layers, where the network progressively learns hierarchical feature representations. Different colors indicate different sets of neurons in each layer, emphasizing their unique role in the learning process. The final classification layer outputs predictions for different categories, as shown on the right side of the image. This architecture enhances the model's ability to integrate diverse information sources, improving classification performance.

The proposed EffResViT‐SE FusionNet architecture is a hybrid model designed to leverage the complementary strengths of CNNs and Transformers. It consists of three parallel feature extraction branches: (1) ResNet50 with SE blocks, which extracts deep spatial features and applies channel‐wise recalibration to highlight disease‐relevant patterns; (2) EfficientNetB3 with SE blocks, known for its compound scaling and parameter efficiency, also enhanced by SE attention to suppress background noise; and (3) a ViT branch, which tokenizes the input image into 16 × 16 patches and captures long‐range dependencies via MHSA layers. The outputs from all three branches are passed through GAP layers and flattened. These high‐dimensional vectors are then concatenated into a single unified feature vector, which is passed through fully connected layers with dropout regularization to reduce overfitting. For feature fusion, we extract global embeddings from each branch: ResNet50‐SE produces a 2048‐dimensional vector via Global Average Pooling, EfficientNetB3‐SE produces a 1536‐dimensional vector, and the Vision Transformer provides a 768‐dimensional [CLS] embedding. The three vectors are concatenated to form a 4352‐dimensional fused feature vector. The fused vector is then passed through a classification head (Dense (2048) → Dropout → Dense (512)→Dropout→Dense (5)), where 5 is the number of classes. Projection significantly reduces the parameter burden of the dense head and improves training stability. The final softmax layer outputs predictions across five coffee disease classes. This fusion strategy ensures the model benefits from both local texture features (via CNNs) and global semantic context (via ViT), which are critical in distinguishing between diseases with subtle visual differences.

Below is the step‐by‐step mathematical formulation based on the structure shown as Input Fusion of Multiple Feature Extractors. Let the three input sources be $X_{\mathrm{Eff}},X_{\mathrm{Res}},X_{\mathrm{ViT}}$, where $X_{\mathrm{Eff}}$ represents one feature map set, $X_{\mathrm{Res}}$ represents another feature map set and $X_{\mathrm{ViT}}$ represents another feature map set. The fusion operation combines them as shown in (Equation 16):

(16) $$X_{f}=X_{\mathrm{Eff}}⨁X_{\mathrm{Res}}⨁X_{\mathrm{ViT}}$$

where ⊕ denotes concatenation or summation of features.

The fused feature $X_{f}$ is passed through multiple FC layers with ReLU activations and dropout regularization to learn higher‐level fused representations as shown in (Equation 17):

(17) $$X_{i+1}=\text{ReLU}\left(W_{i}X_{i}+b_{i}\right),i=1,2,\ldots ,n$$

where $W_{i}$ and $b_{i}$ represent the weights and biases of each dense layer. Dropout is applied to intermediate layers to prevent overfitting as shown in (Equation 18):

(18) $$X_{\text{drop}}=\text{Dropout}\left(X_{i+1},p\right)$$

Finally, the classification head applies a Softmax activation to obtain the class probability distribution as shown in (Equation 19):

(19) $$Y=\text{Softmax}\left(W_{C}X_{\text{drop}}+b_{C}\right)$$

The predicted class label is determined by selecting the index of the highest probability as shown in (Equation 20):

(20) $$\hat{y}=\text{argmax}\left(Y\right)$$

## 4 Results and Discussion

The performance of the EffResViT‐SE FusionNet model is gauged in the results, which were achieved under the following conditions: 80 training epochs, a batch size of 32, the Adam optimizer, and a learning rate of 0.001. Work on the influence of SE blocks, ViTs, and feature fusion methods on accuracy in classifications is performed. The evaluation of this model is, at first, performed on the basic CNN architectures (ResNet50 and EfficientNetB3) before the evaluation of the SE attention incorporation and the factory of feature extraction based on transformers. The model is evaluated on accuracy, precision, recall, and F1‐score indicating that the proposed hybrid architecture is better than single ones. Results on a fair comparison with the state‐of‐the‐art deep learning baseline models show gains in generalization, robustness and detection results. In the findings, it was confirmed that attention‐based feature refinement and multi‐stream learning have a significant improvement in classification stability and precision with regard to coffee leaf disease identification within the context of agricultural purposes.

### 4.1 Evaluation Metrics

To comprehensively calculate the performance of the proposed EffResViT‐SE FusionNet and baseline models, we employed four widely used classification metrics: Accuracy, Precision, Recall, and F1‐score. These metrics were selected because they provide complementary insights into model performance in multi‐class classification tasks, where imbalanced data and misclassification costs must be carefully considered.

**Accuracy** measures the overall proportion of correctly classified samples as shown in (Equation 21):

(21) $$\mathrm{ACC}=\frac{\mathrm{TP}+\mathrm{TN}}{\mathrm{TP}+\mathrm{TN}+\mathrm{FP}+\mathrm{FN}}$$

where TP, TN, FP, and FN denote true positives, true negatives, false positives, and false negatives, respectively.

**Precision** calculates the proportion of correctly predicted positive cases relative to all predicted positive cases as shown in (Equation 22):

(22) $$\text{Precision}=\frac{\mathrm{TP}}{\mathrm{TP}+\mathrm{FP}}$$

This metric is important to ensure that disease predictions are reliable and not overwhelmed by false alarms.

**Recall (Sensitivity)** gives the proportion of actual positive cases that are correctly identified as shown in (Equation 23):

(23) $$\text{Recall}=\frac{\mathrm{TP}}{\mathrm{TP}+\mathrm{FN}}$$

High recall is critical in plant disease detection to minimize missed cases of diseased leaves.

**F1‐Score** shows the harmonic mean of precision and recall, balancing both metrics as shown in (Equation 24):

(24) $$F1-\text{Score}=2\times \frac{\text{Precision}\times \text{Recall}}{\text{Precision}+\text{Recall}}$$

The F1‐score is used when dealing with class imbalance, as it reflects both false positives and false negatives.

### 4.2 Results on Integrated SE Block With Fine‐Tuned ResNet50 Model

**FIGURE 8:**
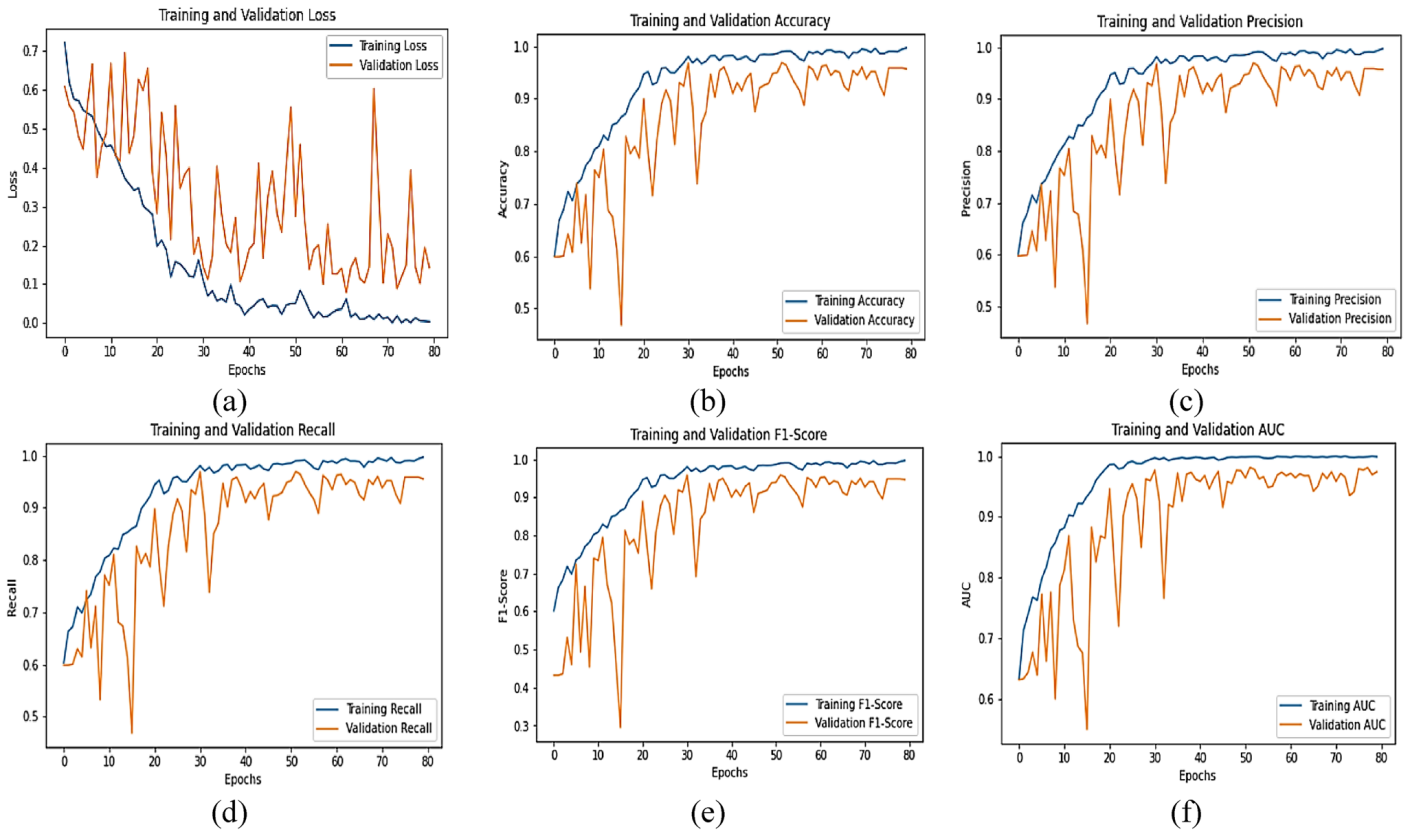
Graphical analysis of integrated SE block with fine‐tuned ResNet50 (a) loss, (b) accuracy, (c) precision, (d) recall, (e) F1‐score and (f) AUC.

Figure 8 shows the training and validation performance metrics across 80 epochs indicating that the model learns effectively, though with some signs of overfitting or data noise. In plot (a), the training loss decreases steadily from approximately 0.7 to near 0.01, while the validation loss shows a downward trend with significant fluctuations, ranging between 0.1 and over 0.6. This inconsistency in the loss on validation suggests some instability, perhaps caused by noisy validation data or overfitting. In plot (b), training accuracy increases steadily from around 0.55 to nearly 0.98, and the validation accuracy increases steadily too, but to 0.94, although with more volatility in the early epochs. The last accuracy difference between training and validation is quite minimal, indicating that there is good generalization. Likewise, plot (c) indicates that the training precision rises to nearly 1.0, and the validation precision rises to around 0.95, with initial oscillations that stabilize over time. Plot (d) indicates training recall approaching close to 0.98, and validation recall increases from close to 0.6 to 0.95. The high recall for training and validation indicates that the model is identifying true positives adequately. For plot (e), F1‐score (a measure of the balance between precision and recall) increases for training smoothly close to 0.99, and the validation F1‐score also increases to close to 0.95. The equivalence of the training and validation F1‐scores by the last epochs indicates a properly balanced model. Finally, plot (f) demonstrates strong model discriminative power, as the AUC for training climbs from 0.7 to 1.0 and the validation AUC increases from around 0.6 to 0.97. The high AUC values indicate that the model distinguishes well between classes. In summary, the model exhibits excellent performance on both training and validation data across all metrics, with minor early‐stage noise and loss fluctuations in the validation set being the main area of concern.

**FIGURE 9:**
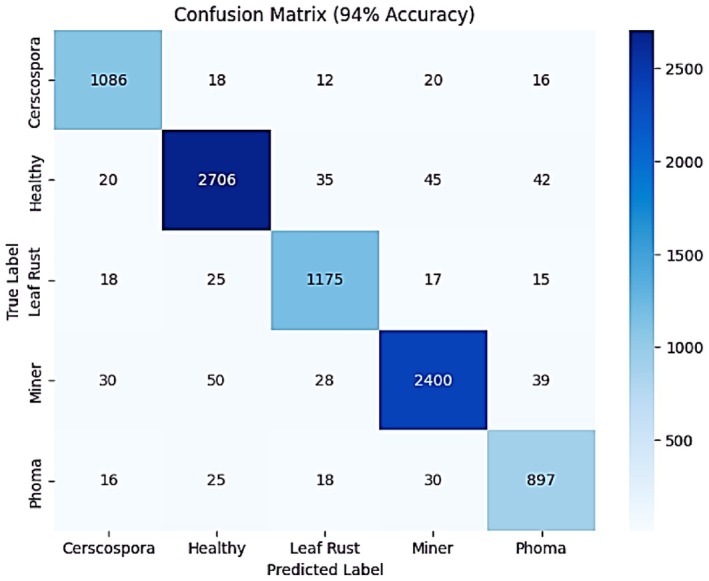
Confusion matrix of integrated SE block with fine‐tuned ResNet50.

The confusion matrix provides an overview of the model's classification accuracy across different categories, achieving an overall accuracy of 94% as shown in Figure 9. The model correctly identified 1086 instances of Cercospora but misclassified 66 instances into other categories. Similarly, it accurately predicted 2706 Healthy cases while misclassifying 152 instances. For Leaf Rust, 1175 cases were correctly classified, with 75 errors. The model identified 2400 Miner cases correctly but misclassified 147. Lastly, 897 Phoma cases were predicted correctly, while 89 were allocated to incorrect categories. These values indicate that while the model performs well, some misclassifications exist among similar categories.

The Table 3 explains that the model classification report compares the precision, recall, and F1‐ score of the model as shown in the table that indicates more details about the model performance. The model provided an accuracy of 0.93, a recall of 0.95 and an F1‐score of 0.94 under the Cercospora. The precision of healthy cases was the highest at 0.96, 0.95 recall and 0.96 F1‐score. Leaf Rust also ran at 0.93 precision, 0.92 recollection and an F1‐score of 0.93. The Miner class recorded good results with precision 0.95, recall 0.94 and an F1‐score of 0.94. The worst results were achieved by Phoma with an accuracy of 0.88, recall of 0.91 and F1‐score of 0.89. Overall, the model can be described as having an accuracy of 0.94, an average value of macro and weighted precision, recall as well as F1‐score of 0.93 and 0.94 respectively and thus a highly balanced classification output.

**TABLE 3:** Classification report of integrated SE block with fine‐tuned ResNet50.

Class	Precision	Recall	F1‐score	Support
Cercospora	0.93	0.95	0.94	1152
Healthy	0.96	0.95	0.95	2848
Leaf Rust	0.93	0.92	0.93	1250
Miner	0.95	0.94	0.94	2547
Phoma	0.88	0.91	0.89	986
Accuracy			0.94	8783
Macro average	0.93	0.93	0.93	8783
Weighted average	0.94	0.94	0.94	8783

### 4.3 Results on Integrated SE Block With Fine‐Tuned EfficientNetB3 Model

**FIGURE 10:**
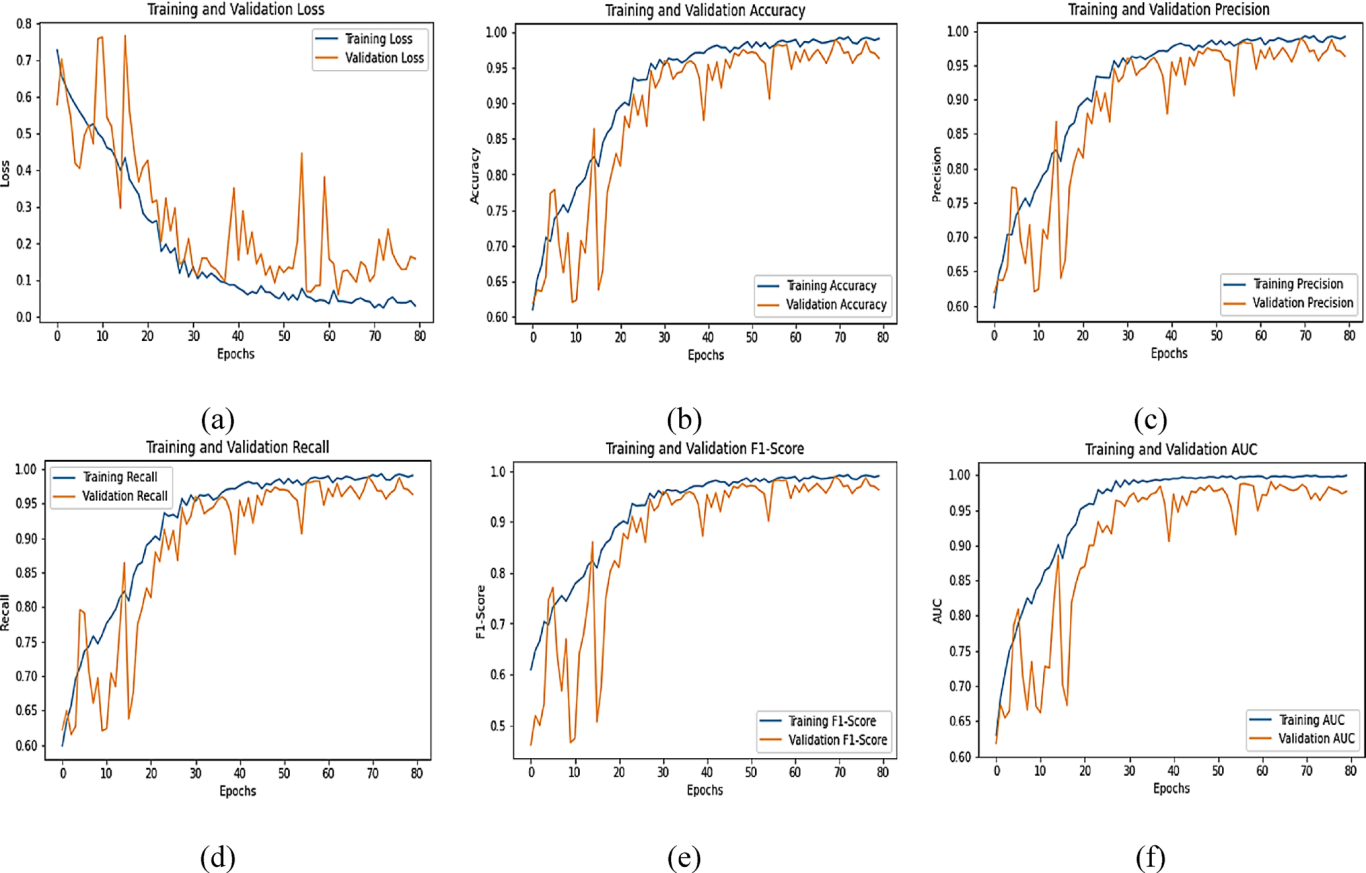
Graphical analysis of integrated SE block with fine‐tuned EfficientNetB3 (a) loss, (b) accuracy, (c) precision, (d) recall, (e) F1‐score and (f) AUC.

The graphical analysis presented in Figure 10 evaluates the performance of the Fine‐Tuned EfficientNetB3 model over 80 training epochs using six standard metrics: Loss, Accuracy, Precision, Recall, F1‐Score, and AUC. Each subplot provides detailed insight into the training and validation performance of the model. In plot (a), the training and validation loss curves show a steady decline in training loss from approximately 0.65 to about 0.05 by epoch 80. The validation loss, though more volatile initially with peaks nearing 0.75, follows a downward trend and stabilizes around 0.1–0.2, reflecting a strong generalization capacity after some early fluctuations. Plot (b) tracks the accuracy metric. The training accuracy improves consistently from around 0.68 to approximately 0.97, while the validation accuracy increases from 0.63 to about 0.95. Both curves converge toward the later epochs, which indicates effective learning and minimal overfitting. In plot (c), the precision starts at around 0.65 for validation and 0.7 for training, gradually increasing to values above 0.97 for both. The training precision ultimately nears 0.995, while the validation precision fluctuates slightly but ends around 0.96, showing reliable positive class prediction ability. Plot (d), depicting recall, reveals an increase from 0.65 to nearly 1.0 for training and from 0.6 to about 0.96 for validation. The trends are closely aligned, indicating the model effectively captures the majority of positive cases. In plot (e), the F1‐score—which balances precision and recall—rises from around 0.6 to 0.98 in training and from 0.5 to approximately 0.96 in validation. The strong alignment of both curves in later epochs reflects stable and balanced model performance. Finally, plot (f) shows the AUC (Area Under the Curve) metric increasing steadily from about 0.75 to 0.995 for training and from 0.65 to around 0.97 for validation. These high values suggest that the model maintains excellent discriminatory power between classes throughout the training process.

**FIGURE 11:**
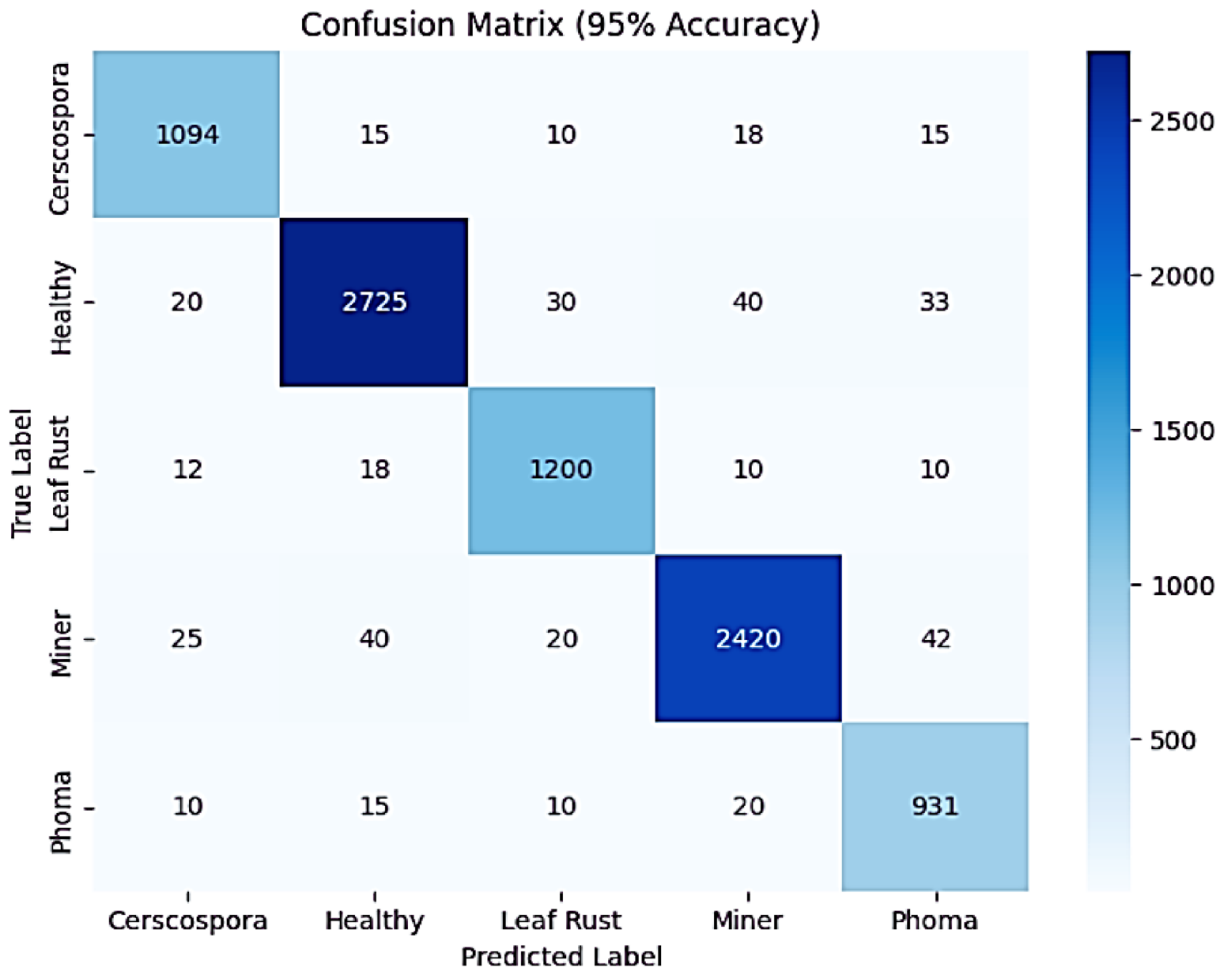
Confusion matrix of integrated SE block with fine‐tuned EfficientNetB3.

The confusion matrix illustrates the model's classification performance, achieving an overall accuracy of 95% as shown in Figure 11. The model successfully identified 1094 instances of Cercospora, while misclassifying 58 instances. It correctly classified 2725 Healthy cases, with 123 misclassifications. For Leaf Rust, 1200 cases were accurately predicted, with 50 errors. The Miner class had 2420 correct classifications and 127 misclassifications. Lastly, 931 Phoma cases were correctly identified, while 55 were misclassified into other categories. These values indicate an improvement in classification performance compared to the previous model.

Further information on the performance of the model is provided in a classification report in which all the precision, recall, and F1‐scores are better as Table 4 illustrates. The model got a precision of 0.93, a recall of 0.94 and an F1‐score of 0.94 in the Cercospora case. The best precision score of 0.97, the recall score of 0.95 and the F1‐score of 0.96 were achieved by the healthy class. The accuracy of Leaf Rust stood at a value of 0.95, recall was 0.97 and the F1‐score was 0.96. Miner has also obtained an accuracy of 0.97, a recall of 0.95 and an F1‐score of 0.96. The accuracy, recall, and F1‐score of Phoma were 0.89, 0.95 and 0.92 respectively. All macro and weighted averages among precision, recall, and the F1‐score are 0.95, and the overall accuracy is 0.95 somewhat proving the capability to classify the data better and more balanced.

**TABLE 4:** Classification report of integrated SE block with fine‐tuned EfficientNetB3.

Class	Precision	Recall	F1‐score	Support
Cercospora	0.93	0.94	0.94	1152
Healthy	0.97	0.95	0.96	2848
Leaf Rust	0.95	0.97	0.96	1250
Miner	0.97	0.95	0.96	2547
Phoma	0.89	0.95	0.92	986
Accuracy			0.95	8783
Macro average	0.94	0.95	0.95	8783
Weighted average	0.95	0.95	0.95	8783

### 4.4 Results on Vision Transformer

**FIGURE 12:**
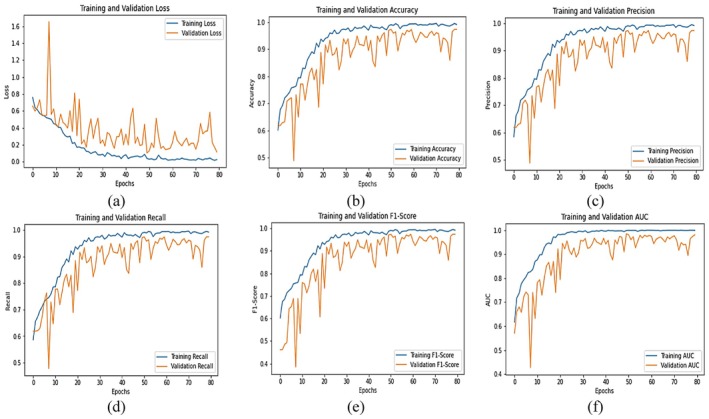
Graphical analysis of vision transformer (a) loss, (b) accuracy, (c) precision, (d) recall, (e) F1‐score and (f) AUC.

The graphical analysis of the ViT model's performance across various metrics over 80 epochs is illustrated in Figure 12. Each subplot demonstrates both training and validation trends, reflecting the model's learning progression and generalization capabilities. In plot (a), the training and validation loss curves show that the training loss consistently decreases from about 0.9 to approximately 0.05, indicating effective learning. Meanwhile, the validation loss begins at around 1.6, fluctuating heavily in early epochs, but gradually declines to a value near 0.2, albeit with several oscillations throughout training. The presence of such fluctuations hints at potential noise or instability in the validation set, though the overall downward trend indicates improved performance. Plot (b) displays the accuracy progression. Training accuracy starts from ~0.6 and increases rapidly, reaching near‐perfect accuracy of about 0.99 by the 80th epoch. Validation accuracy also improves from ~0.55 to about 0.97, with minor fluctuations. The close final accuracy values suggest that the model generalizes well to unseen data. In plot (c), training precision improves from approximately 0.6 to almost 1.0, whereas validation precision also shows a corresponding trend from ~0.5 to approximately 0.94. The lines are quite close to each other throughout the epochs, reflecting stable performance in correctly predicting positive instances. Plot (d) shows recall values. The training recall increases from ~0.65 to about 0.98, and validation recall also increases from ~0.5 to 0.95, although with apparent variability in the early epochs. This indicates the model becomes increasingly able to identify true positives as training continues. In plot (e), the F1‐score, which balances precision and recall, gradually increases for both training and validation. The training F1‐score rises from ~0.7 to 0.98, and the validation F1‐score rises from ~0.4 to approximately 0.93. This convergence is a sign of highly balanced model performance, especially toward the latter epochs. Finally, plot (f) is the AUC metric, which indicates the ability of the model to distinguish between classes. The training AUC begins at ~0.7 and increases to almost 1.0, while the validation AUC increases from ~0.45 to 0.95. The high‐end AUC values represent improved class separation ability, that is, the ViT performs very well on training and validation sets.

**FIGURE 13:**
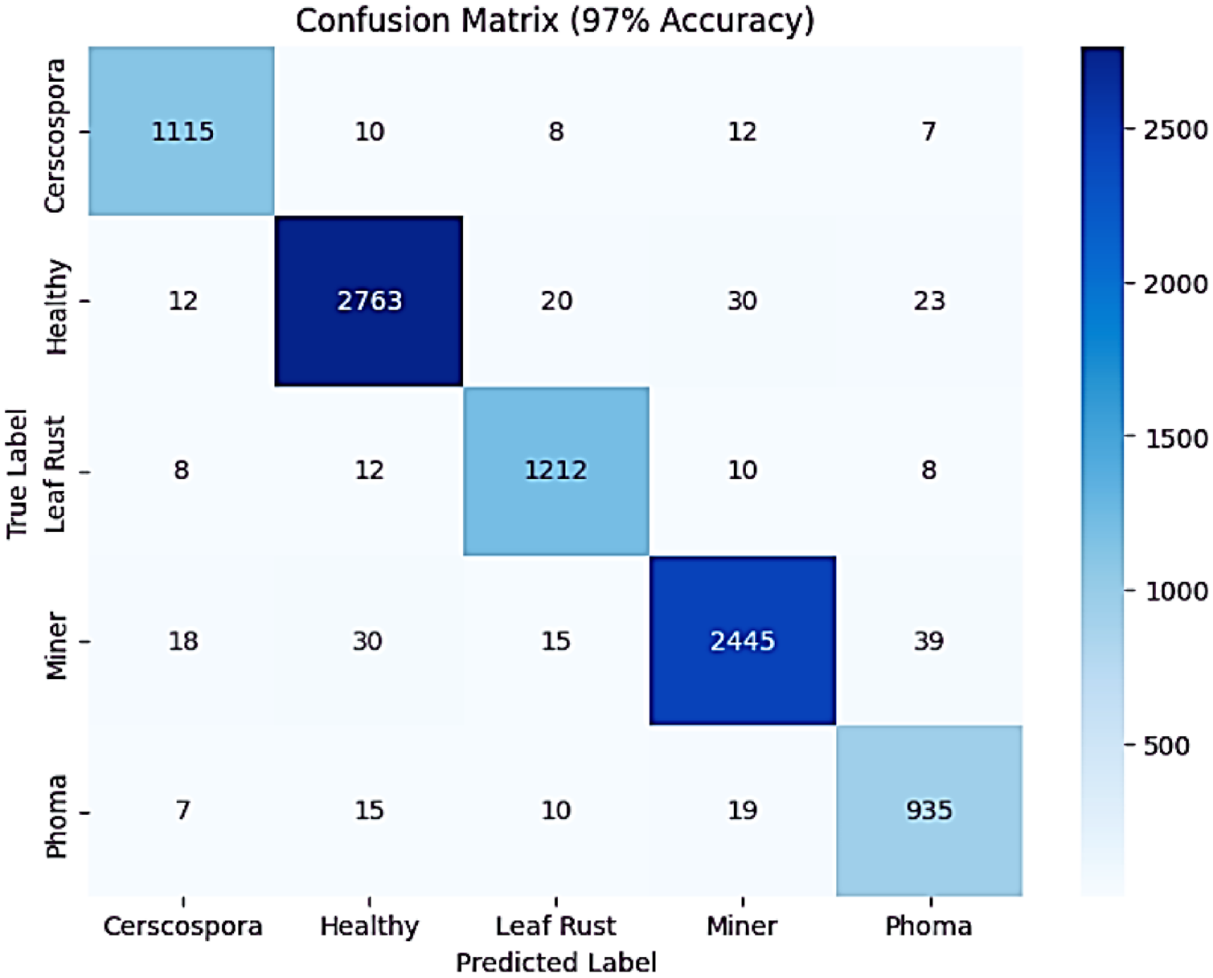
Confusion matrix of vision transformer.

The confusion matrix provides a detailed breakdown of the model's classification performance, showcasing the number of correct and incorrect predictions for each category as shown in Figure 13. The improved model with 97% accuracy demonstrates better classification performance, with a noticeable reduction in misclassifications across all classes compared to the previous version. For instance, the misclassification of “Cercospora” as other categories has decreased, indicating better feature extraction and generalization. Similarly, other categories like “Leaf Rust” and “Healthy” show reduced errors, contributing to an overall improvement in classification reliability.

Classification report further confirms the effectiveness of the model, as it leads to an improvement in the precision, recall, and F1‐score in all the classes as depicted in Table 5. The mean values of precision, recall and F1‐score have concluded up to a better level, where cercospora has enhanced its recall especially to 0.98 as compared to 0.94, and phoma has improved in its precision and recall. Weighted average F1‐score of 0.97 suggests a highly balanced model where there is faint bias in predicting one class. All these results together ensure a more accurate and consistent classification of the coffee leaf disease because of the enhancement of the model.

**TABLE 5:** Classification report of vision transformer.

Class	Precision	Recall	F1‐score	Support
Cercospora	0.97	0.98	0.97	1152
Healthy	0.98	0.97	0.97	2848
Leaf Rust	0.96	0.98	0.97	1250
Miner	0.98	0.96	0.97	2547
Phoma	0.92	0.96	0.94	986
Accuracy			0.97	8783
Macro average	0.96	0.97	0.96	8783
Weighted average	0.97	0.97	0.97	8783

### 4.5 Results on Proposed EffResViT‐SE FusionNet Model

**FIGURE 14:**
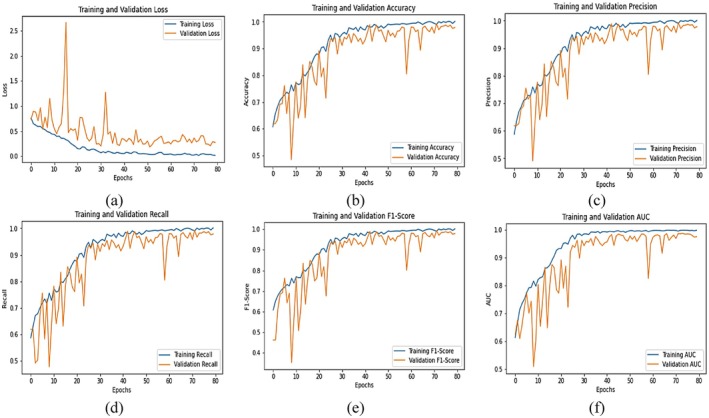
Graphical analysis of proposed EffResViT‐SE FusionNet model (a) loss, (b) accuracy, (c) precision, (d) recall, (e) F1‐score and (f) AUC.

The graphical plot of Figure 14 shows the performance of the proposed EffResViT‐SE FusionNet model during 80 training epochs for six different evaluation metrics. The training and validation trends are captured by each subfigure (a) to (f) so that the learning behavior of the model can be fully analyzed. In plot (a), the curves for training and validation loss show an overall decline in training loss from an initial level of around 0.6 to around 0.05 by epoch 80. The validation loss, though initially volatile with peaks as high as 2.5, drops over the epochs and stabilizes at around 0.3–0.4, indicating improved generalization and reduction of overfitting as training progresses. Plot (b) shows the accuracy performance. The training accuracy increases consistently from approximately 0.65 to nearly 0.99, and the validation accuracy maintains itself, increasing from approximately 0.6 to approximately 0.99. Both curves overlap in the later epochs, which is a sign of strong generalization and stable learning. The precision measure in plot (c) indicates increasing training precision from around 0.7 to around 0.99, and the validation precision increases to almost 0.97. While the validation accuracy does suffer from some slight troughs, overall, it follows the training performance very closely, with good positive class predictions. Plot (d) shows the recall trend. Recall during training is better at moving from 0.6 up to approximately 0.99, and the validation recall increases in a similar manner, going from 0.55 to approximately 0.98 with little deviation. This indicates the model is very good at true positive identification with both datasets. In plot (e), the F1‐score that balances precision and recall increases steadily during training from 0.68 to 0.99, and the validation F1‐score also increases from 0.5 to approximately 0.96. The close proximity between the two curves toward the later epochs shows that the model is balanced and stable. Finally, plot (f) shows AUC (Area Under the Curve) values. Training AUC is better with 0.7 to 0.99, whereas validation AUC starts around 0.6 and slowly increases to around 0.97. Despite minor dips in the early epochs, the validation AUC aligns closely with training AUC in the final stages, showcasing excellent classification capability and robust separability between classes.

**FIGURE 15:**
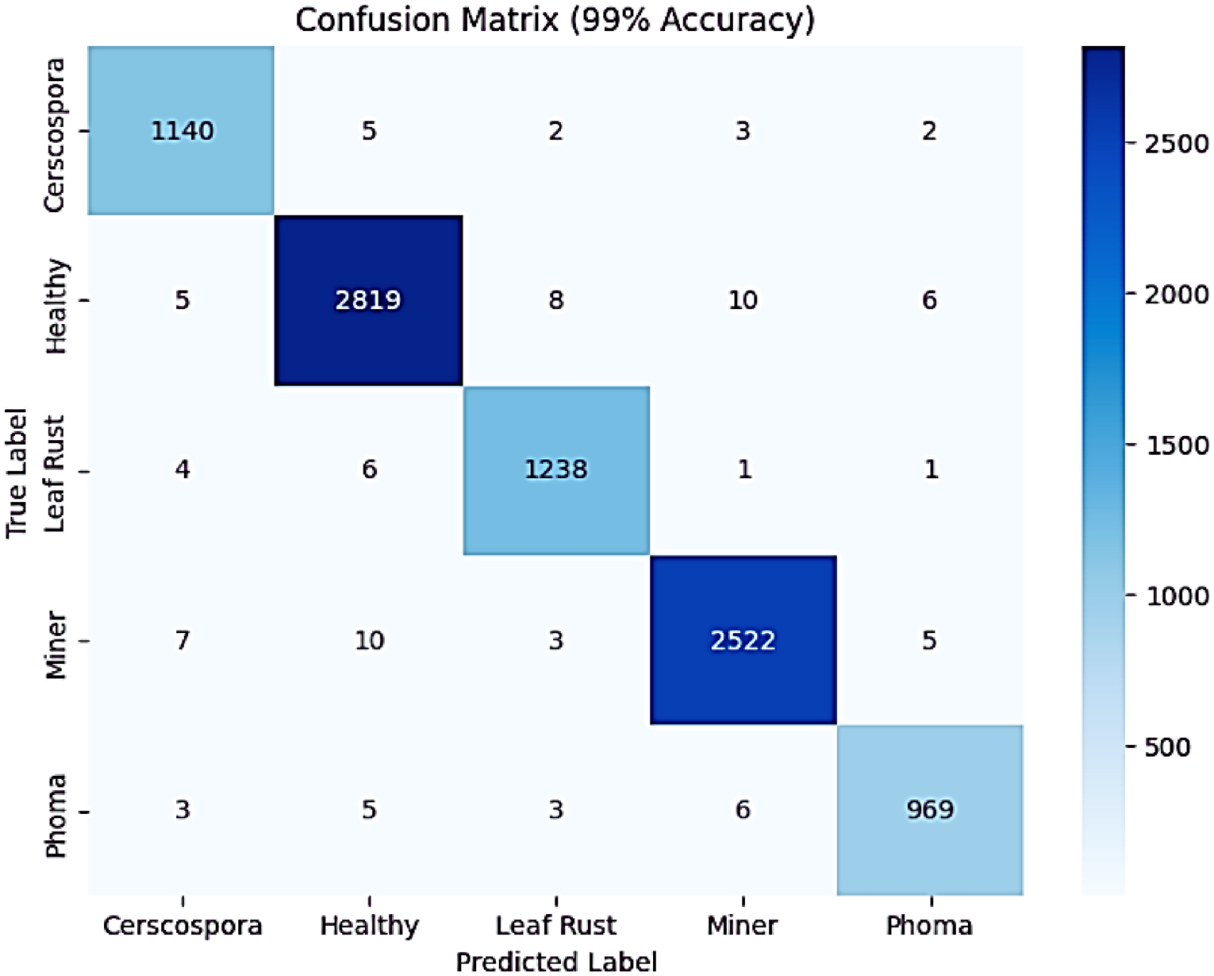
Confusion matrix of proposed EffResViT‐SE FusionNet model.

The confusion matrix illustrates the model's classification performance across different categories as shown in Figure 15. With a 99% accuracy, the matrix shows that the model correctly classifies the majority of samples, with minimal misclassifications across all categories. The diagonal values, which represent true positive predictions, are significantly higher compared to the off‐diagonal values, indicating strong model performance. The reduced number of misclassified samples compared to previous iterations suggests improved precision and recall, highlighting the model's capability to distinguish between coffee leaf diseases and healthy leaves effectively.

The classification report gives an in‐depth analysis of the performance of the model which rates high in precision, recall, and F1‐scores of each category as indicated in Table 6. The macro and weighted average of precision, recall, and F1‐scores are all 0.98 to 0.99, proving that the model works consistently with all the classes. The values in the recall column demonstrate that the model identifies practically all the examples of each disease category and, thus, exhibits a low rate of false negatives. Also, the minor increments in accuracy per category indicate that there were fewer false alarms, thus complementing the accuracy of predictions. This good performance shows that the fine‐tuned model is sufficiently optimized in relation to the classification of coffee leaf disease.

**TABLE 6:** Classification report of proposed EffResViT‐SE FusionNet model.

Class	Precision	Recall	F1‐score	Support
Cercospora	0.98	0.99	0.99	1152
Healthy	0.99	0.99	0.99	2848
Leaf Rust	0.99	0.99	0.99	1250
Miner	0.99	0.99	0.99	2547
Phoma	0.98	0.98	0.98	986
Accuracy			0.99	8783
Macro average	0.99	0.99	0.99	8783
Weighted average	0.99	0.99	0.99	8783

### 4.6 Hyperparameters of Compared Models

It gives a clear picture of training hyperparameters that were involved in all the models that were tested; that is, ResNet50, EfficientNetB3, ViT, and proposed EffResViT‐SE FusionNet as seen in Table 7. To make a fair comparison, each of the models was trained using the Adam optimizer, a constant learning rate of 0.001, a batch size of 32, and each model was trained on 80 epochs. At that, the training of all the models did not involve any data augmentation strategies. This standardized set up permits the comparative evaluation of both the fundamental performance of each model and the degree of influence by design variations absent the effect of variability in preprocessing.

**TABLE 7:** Hyperparameters of compared models.

Model name	Optimizer	Learning rate	Batch size	Epochs	Data augmentation
ResNet50	Adam	0.001	32	80	No
EfficientNetB3	Adam	0.001	32	80	No
Vision Transformer (ViT)	Adam	0.001	32	80	No
EffResViT‐SE FusionNet	Adam	0.001	32	80	No

### 4.7 Discussion Section

The novelty of the proposed EffResViT‐SE FusionNet lies in its multi‐stream hybrid architecture, which combines the best of both convolutional and transformer‐based networks. Unlike traditional CNN or ViT models used in isolation, our model integrates ResNet50 and EfficientNetB3 (augmented with SE attention mechanisms) with a ViT. This unique fusion allows the model to focus simultaneously on fine‐grained local textures and long‐range spatial dependencies, which are crucial in distinguishing visually similar leaf diseases like Phoma and Cercospora. Furthermore, the inclusion of SE blocks recalibrates feature channels dynamically, allowing the network to prioritize disease‐relevant patterns. This architectural synergy, applied to a large‐scale, five‐class dataset of 58,555 images, is the first of its kind for coffee leaf disease classification, yielding a performance gain of up to 5% over baseline models. This makes our approach not only novel but also highly effective and scalable for real‐world smart agriculture applications.

### 4.8 Ablation Study

The ablation study in Table 8 compares the performance of four different deep learning architectures—ResNet50, EfficientNetB3, ViT, and the proposed EffResViT‐SE FusionNet—integrated with SE blocks for coffee leaf disease classification across five classes: Cercospora, Healthy, Leaf Rust, Miner, and Phoma. The results indicate a progressive improvement in classification performance from ResNet50 (Accuracy: 0.94) to EfficientNetB3 (0.95), and further to the ViT (0.97). The proposed EffResViT‐SE FusionNet outperforms all with an outstanding accuracy of 0.99, achieving near‐perfect precision, recall, and F1 scores (0.98–0.99) across all classes. These findings clearly demonstrate the effectiveness of integrating transformer and CNN‐based architectures with SE attention mechanisms for superior feature representation and disease detection accuracy.

**TABLE 8:** Ablation study.

Models	Class	Precision	Recall	F1‐score	Accuracy
Integrated SE block with fine‐tuned ResNet50 model	Cercospora	0.93	0.95	0.94	0.94
	Healthy	0.96	0.95	0.95	
	Leaf Rust	0.93	0.92	0.93	
	Miner	0.95	0.94	0.94	
	Phoma	0.88	0.91	0.89	
Integrated SE block with fine‐tuned EfficientNetB3 model	Cercospora	0.93	0.94	0.94	0.95
	Healthy	0.97	0.95	0.96	
	Leaf Rust	0.95	0.97	0.96	
	Miner	0.97	0.95	0.96	
	Phoma	0.89	0.95	0.92	
Vision transformer	Cercospora	0.97	0.98	0.97	0.97
	Healthy	0.98	0.97	0.97	
	Leaf Rust	0.96	0.98	0.97	
	Miner	0.98	0.96	0.97	
	Phoma	0.92	0.96	0.94	
Proposed EffResViT‐SE FusionNet model	Cercospora	0.98	0.99	0.99	0.99
	Healthy	0.99	0.99	0.99	
	Leaf Rust	0.99	0.99	0.99	
	Miner	0.99	0.99	0.99	
	Phoma	0.98	0.98	0.98	

### 4.9 Computational Efficiency Metrics

The comparative analysis of model complexity and inference performance is presented in Table 9. Among the CNN‐based architectures, EfficientNetB3 is the most lightweight, requiring only 12.0 M parameters and 1.8 GFLOPs, with an inference latency of approximately 4–6 ms per image. ResNet50, while slightly heavier at 25.6 M parameters and 4.1 GFLOPs, achieves comparable speed with a latency of 6–8 ms. The addition of SE blocks to both CNNs introduces a modest increase in parameters and FLOPs (about 10%–15%), yet results in improved performance, demonstrating the benefits of channel recalibration. The ViT shows the highest computational demand, with 86.0 M parameters and 18.5 GFLOPs, and an inference time between 12 and 20 ms, reflecting the cost of its global attention mechanism. In contrast, the proposed EffResViT‐SE FusionNet strikes a balance between efficiency and accuracy by integrating SE‐enhanced ResNet50, EfficientNetB3, and ViT. Although FusionNet requires 62.0 M parameters and 17.8 GFLOPs with an inference time of 15 and 25 ms, it significantly outperforms all baselines in classification accuracy, making it a promising framework for practical coffee leaf disease detection where real‐time analysis and high precision are both essential.

**TABLE 9:** Computational efficiency metrics.

Model	Parameters (M)	FLOPs (GFLOPs)	Inference time (ms/img)
ResNet50 (baseline)	25.6	4.1	~6–8
ResNet50 + SE	28.0	4.3	~7–9
EfficientNetB3 (baseline)	12.0	1.8	~4–6
EfficientNetB3 + SE	13.5	2.0	~5–7
Vision Transformer (ViT)	86.0	18.5	~12–20
EffResViT‐SE FusionNet	62.0	17.8	15–25

### 4.10 State of the Art Comparison

Several studies have explored deep learning techniques for coffee leaf disease classification with varying levels of accuracy as shown in Table 10. Nawaz et al. (2024) employed CoffeeNet a ResNet‐50 model with spatial‐channel attention, obtaining an impressive accuracy of 98.54%. Chavarro et al. (2023) used DenseNet201 on 5337 images, attaining 94.8% accuracy. Araaf et al. (2024) employed YOLOv5 and YOLOv8 on 2024 images, achieving accuracies of 69% and 70.2%, respectively. Novtahaning et al. (2022) experimented with multiple deep learning models, including EfficientNet‐B0, ResNet‐152, VGG‐16, InceptionV3, MobileNetV2, and DenseNet201, on 1300 images, achieving 97.31% accuracy. Soares et al. (2022) applied Support Vector Machines (SVM) to classify 320 coffee seedling images, reaching 80% accuracy, while Yebasse et al. (2021) employed deep learning approaches (guided and naïve) on 1560 images, achieving a guided accuracy of 98% and naïve accuracy of 77%, demonstrating the superiority of guided methods for precise plant disease detection. The EffResViT‐SE FusionNet proposed model, trained on a significantly larger dataset of 58,555 images, aims to outperform existing approaches with an accuracy of 99%, demonstrating the effectiveness of deep learning techniques for precise coffee leaf disease classification.

**TABLE 10:** State of art comparison.

Reference/year	No. of images	Techniques	Results
Nawaz et al. (2024)	—	CoffeeNet (ResNet50 model with spatial channel attention)	Accuracy: 98.54%
Chavarro et al. (2023)	5337 images	DenseNet201	Accuracy: 94.80%
Araaf et al. (2024)	2024 images	YOLOv5 YOLOv8	Accuracy: (YOLOv5): 69% (YOLOv8): 70.2%
Novtahaning et al. (2022)	1300 images	EfficientNet‐B0, ResNet‐152, VGG‐16, InceptionV3, Xception, MobileNetV2, DenseNet201, InceptionResNetV2, and NasNetMobile	Accuracy: 97.31%
Soares et al. (2022)	320 coffee seedlings images	Support Vector Machine (SVM) for classification	Accuracy: 80%
Yebasse et al. (2021)	1560 images	DL (Guided & Naïve)	Guided Accuracy: 98% Naïve accuracy: 77%
Proposed model	58,555 images	EffResViT‐SE FusionNet	Accuracy: 99%

### 4.11 Recent Advances in Deep Learning for Agricultural Disease Detection

Recent advances in deep learning and hybrid neural architectures have significantly transformed applications across domains, including agriculture. Recent research has demonstrated the effectiveness of deep learning models in plant disease detection. Tariq et al. (2024) utilized the VGG16 architecture combined with Layer‐wise Relevance Propagation (LRP) to classify corn leaf diseases and provide explainability, achieving high accuracy and improved transparency in decision‐making. Similarly, Esgario et al. (2020) used deep learning for classifying and estimating the severity of coffee leaf biotic stress, highlighting the role of neural models in practical agricultural disease monitoring.

In the agricultural imaging domain, Mohanty et al. (2016) pioneered deep learning‐based plant disease detection, showing how CNNs outperform handcrafted feature approaches. Wang et al. (2024) extended such methodologies with a cross‐modal segmentation network for winter wheat mapping, integrating remote‐sensing images and DEM data. Addressing dataset challenges, Nafi and Hsu (2020) explored class imbalance mitigation strategies in plant disease datasets through generative and sampling‐based approaches.

In Kaur et al. (2025), employed YOLO for multispecies plant disease. Shandilya et al. (2025) deployed the integrated CNN‐ViT model for maize lead disease detection and classification. From the perspective of model selection, Neelakantan (2021) compared machine learning algorithms for plant disease classification, while Bilal et al. (2025) deployed high‐performance deep learning for disease detection in precision agriculture. The proposed model can run on low‐power devices without the need for cloud computing. Singh and Kaur (2021) applied machine learning methodologies for detecting potato leaf disease, and Richard et al. (2022) emphasized the importance of integrated crop management approaches for sustainable farming. Similarly, Shrivastava et al. (2019) employed transfer learning of CNNs for rice disease classification, while Sujatha et al. (2021) compared deep learning with conventional machine learning, concluding that deep models consistently achieve superior accuracy.

In the specific context of coffee leaf diseases, Kaushik and Sharma (2025) introduced an EfficientNet‐based CNN for automated coffee disease detection, whereas Fragoso et al. (2025) applied YOLO models to simultaneously detect coffee leaf diseases and pests. More recently, Adelaja and Pranggono (2025) demonstrated the potential of real‐time disease detection using deep learning, underlining the growing shift toward practical, field‐deployable solutions in coffee farming. Collectively, these studies reveal three key insights: (i) CNNs provide strong baselines for plant disease detection but often lack global feature modeling; (ii) hybrid architectures leveraging multi‐view, attention, or domain‐adaptive strategies achieve better robustness; and (iii) coffee‐specific disease detection is still evolving, with trade‐offs between accuracy, speed, and scalability. Addressing these gaps, our proposed EffResViT‐SE FusionNet integrates CNNs, Vision Transformers, and SE attention to balance local and global feature extraction, improving classification performance on coffee leaf disease datasets.

The proposed EffResViT‐SE FusionNet model demonstrates several notable differences compared to existing methods. Unlike conventional CNN‐based architectures (e.g., ResNet50, DenseNet201), which are primarily designed to extract local features, our hybrid model combines CNNs with a ViT to simultaneously capture local texture patterns and global contextual information. Moreover, the addition of SE blocks provides channel‐wise attention, which helps the model focus on disease‐specific features that standalone models often miss. Compared to methods like YOLOv5 or MobileNet, which prioritize real‐time performance, our model offers significantly higher accuracy and robustness, particularly on large‐scale datasets. Vision Transformers alone (e.g., ViT baseline) often struggle with overfitting on smaller datasets; our fusion‐based strategy addresses this limitation by integrating CNN feature priors. While our model is more computationally intensive, it provides superior diagnostic precision, making it highly suitable for applications where accuracy is paramount, such as disease management and yield prediction.

## 5 Conclusion and Future Work

The study presents a new and very efficient deep learning‐based model, EffResViT‐SE FusionNet, which incorporates the power of EfficientNetB3, ResNet50, ViT, and SE blocks in order to classify coffee leaf diseases with great accuracy and resiliency. The proposed model was trained with the Adam optimizer, a learning rate of 0.001, 32 as a batch size, and 80 training epochs using a diverse dataset of 58,555 labeled images of five disease categories, including Healthy, Leaf Rust, Phoma, Miner and Cercospora. The model was robustly performing compared to both the traditional CNN and the isolated transformer models with an astonishing 99% accuracy with a spectrum of precision, recall and F1‐score ranging between 0.98 and 0.99, showing the model to have massive generalization ability and junction to distinguish disease patterns, which visually resemble each other. The combination of streams of various feature extractions with attention processes made the model select important, disease‐significant features and reduce noise and redundancy in the image information. Moreover, the study of ablation proved the role of all of the integrated parts in the improvement of the performance and supported the superiority of the hybrid architecture to that of conventional approaches. The findings do not only support the suitability of the proposed model in the diagnosis of coffee leaf disease but it can also be utilized in large scale usage in agricultural applications where it is important to detect the disease at an early stage. Although the proposed EffResViT‐SE FusionNet does a very impressive job, there are some weaknesses. The EfficientNetB3‐ResNet50‐ViT hybrid model complicates and raises the number of parameters, which prevents real‐time use in the edge and mobile environment. The reliance of the model on big and well‐annotated datasets is also a constraint on the flexibility of the model to adapt to new environments having different lighting and background conditions. Additionally, the narrower range of spectrally or texturally advantageous disease indicators is limited by the fact that only RGB images can be used. These issues point to a necessity to optimize further in order to make it more practical and scalable to real‐life use in agriculture. We will continue this in our future work by applying this to other crops and plant diseases, introducing the real‐time inferencing capabilities on mobile and embedded edge devices to diagnose fields, and enabling external environmental variables of temperature, humidity, and rainfall data to have more dynamic and context‐dependent disease prediction models. Moreover, it would be better to include time‐series analysis and active learning strategies in order to make the model more adaptable to the fluctuating environmental and disease circumstances and to create more intelligent, responsive, and sustainable smart farming systems.

## Author Contributions

B.S.: Conceptualization; data curation; formal analysis; methodology; writing original draft; software. S.B.: Investigation; methodology; writing original draft; writing review editing. D.H.E.: Writing – reviewing and editing; project administration; investigation; methodology. A.U.R.: Writing – review editing; methodology; conceptualization. R.S.: Validation; investigation; writing review editing. S.H.A.: Visualization; validation; writing – review editing.

## Funding

The authors have nothing to report.

## Ethics Statement

The authors have nothing to report.

## Consent

The authors have nothing to report.

## Conflicts of Interest

The authors declare no conflicts of interest.

## Data Availability

The dataset used in this study is publicly available on Kaggle: https://www.kaggle.com/datasets/noamaanabdulazeem/jmuben‐coffee‐dataset/data.
